# The genome sequence of *Propionibacterium acidipropionici* provides insights into its biotechnological and industrial potential

**DOI:** 10.1186/1471-2164-13-562

**Published:** 2012-10-19

**Authors:** Lucas P Parizzi, Maria Carolina B Grassi, Luige A Llerena, Marcelo F Carazzolle, Verônica L Queiroz, Inês Lunardi, Ane F Zeidler, Paulo JPL Teixeira, Piotr Mieczkowski, Johana Rincones, Gonçalo AG Pereira

**Affiliations:** 1 Laboratório de Genômica e Expressão, Departamento de Genética e Evolução, Instituto de Biologia, Universidade Estadual de Campinas, CP 6109, Campinas, 13083-970, São Paulo, Brazil; 2 Braskem S.A, CP 6192, Campinas, 13083-970, São Paulo, Brazil; 3 Department of Genetics, School of Medicine, Carolina Center for Genome Sciences, University of North Carolina, Wilson Hall, Rm 341, CB#3280, Chapel Hill, NC, 27599-3280, USA

**Keywords:** Genome, *Propionibacterium acidipropionici*, Propionic acid, Biotechnology

## Abstract

**Background:**

Synthetic biology allows the development of new biochemical pathways for the production of chemicals from renewable sources. One major challenge is the identification of suitable microorganisms to hold these pathways with sufficient robustness and high yield. In this work we analyzed the genome of the propionic acid producer Actinobacteria *Propionibacterium acidipropionici* (ATCC 4875).

**Results:**

The assembled *P. acidipropionici* genome has 3,656,170 base pairs (bp) with 68.8% G + C content and a low-copy plasmid of 6,868 bp. We identified 3,336 protein coding genes, approximately 1000 more than *P. freudenreichii* and *P. acnes*, with an increase in the number of genes putatively involved in maintenance of genome integrity, as well as the presence of an invertase and genes putatively involved in carbon catabolite repression. In addition, we made an experimental confirmation of the ability of *P. acidipropionici* to fix CO_2_, but no phosphoenolpyruvate carboxylase coding gene was found in the genome. Instead, we identified the pyruvate carboxylase gene and confirmed the presence of the corresponding enzyme in proteome analysis as a potential candidate for this activity. Similarly, the phosphate acetyltransferase and acetate kinase genes, which are considered responsible for acetate formation, were not present in the genome. In *P. acidipropionici*, a similar function seems to be performed by an ADP forming acetate-CoA ligase gene and its corresponding enzyme was confirmed in the proteome analysis.

**Conclusions:**

Our data shows that *P. acidipropionici* has several of the desired features that are required to become a platform for the production of chemical commodities: multiple pathways for efficient feedstock utilization, ability to fix CO_2_, robustness, and efficient production of propionic acid, a potential precursor for valuable 3-carbon compounds.

## Background

A major challenge of white (industrial) biotechnology is the production with high yield of reduced carbon chains able to replace fossil hydrocarbons. Two examples of well-established processes able to produce high volumes of useful carbon chains are ethanol fermentation by yeast and lactate fermentation by lactic acid bacteria. Ethanol is used mainly as a biofuel and more recently as a substrate in polyethylene production [1]. Lactic acid is used mainly in the food industry, but is currently employed as the building block for the biodegradable plastic polylactic acid (PLA) [1].

Though economically viable, both processes have weaknesses. Ethanol production has a low carbon recovery (51% w/w) due to the decarboxylation of pyruvate being a mandatory step for ethanol formation. Carbon recovery in lactate fermentation is much higher (100% w/w from glucose); however lactic acid has a high oxygen content, which makes its conversion into other industrially relevant 3-carbon molecules difficult. For that reason, it is important to search for new fermenting organisms capable of producing reduced carbon chains with higher efficiency in carbon recovery.

**Figure 1:**
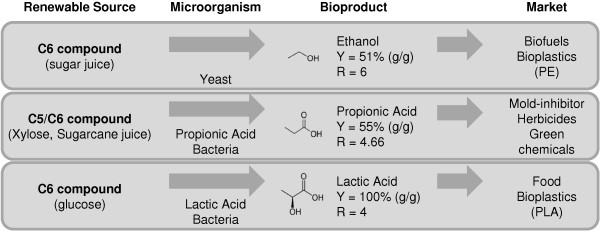
**Overview of well-established fermentation processes.** Comparison between fermentation processes for production of ethanol, propionic acid and lactic acid. Y, theoretical maximum yield from glucose R, reduction factor.

In this context, *Propionibacterium acidipropionici* is a good candidate to be developed for biotechnological processes (Figure 1). *P. acidipropionici* has been widely studied for the heterofermentative production of propionic acid, including fermentation on a semi-industrial scale (10 m^3^) [2]. Propionic acid is more reduced than lactic acid and can be produced with higher yields than ethanol in yeast: a yield of 0.65 g/g of propionic acid was obtained from glucose [3], 0.72 g/g from glycerol and 0.55 g/g from molasses [4]. Propionic acid and its salts are valuable industrial products with several applications such as mold-inhibitors, preservatives for animal and human food, fruit flavorings, essence base, additives in cellulosic plastics, herbicides and medications for animal therapy [5]. Annual world consumption of propionic acid was estimated at 293.4 thousand tonnes in 2009, representing a market of approximately 530 million dollars with an expected growth rate of 3.9% until 2014 [6].

*Propionibacterium* are classified under the high GC division (class Actinobacteria) of bacteria and are overall characterized as Gram-positive, rod-like, pleomorphic, non-spore-forming, non-motile and facultative anaerobic or aerotolerant bacteria. These bacteria are further classified into two groups: cutaneous, found on different areas of human skin; and dairy, isolated mainly from cheese and milk. *P. acidipropionici* is classified in the dairy group. Although differentiated by their typical natural habitats, all species of *Propionibacterium* produce propionic acid as the major fermentative product, with acetic acid and carbon dioxide as the main subproducts [7].

It is noteworthy that even after 100 years of accumulated research on propionibacteria*,* propionic acid is still produced via petrochemical routes and no industrial biotechnology process for these organisms has been established. The main hindrances have been low productivity, low final product concentration, slow growth, high end-product inhibition, and costly downstream separation from sub products [8,9]. To improve propionic acid fermentation by *P. acidipropionici* it is necessary to obtain more detailed information about the organism’s basic biology, especially its molecular biology with only a few studies focusing on the genetics of *P. acidipropionici*[10-12].

Consequently, in this work the molecular biology of *P. acidipropionici* was investigated mainly through genome sequencing and its comparative analysis with three close related and fully sequenced bacteria species. Experimental confirmation of key pathways were performed by a preliminary proteome analysis, fermentation tests with and without 2-Deoxy-D-Glucose and/or C^13^ flux analysis of C^13^O_2_ supplemented fermentation. We identified physiological and metabolic traits that characterize *P. acidipropionici* as a bioreactor for the production of C3 compounds and discuss the possibility of genetically modifying this species to convert propionic acid into more valuable products such as propionaldehyde, n-propanol, acrylic acid and propylene.

## Results and discussion

### General genome features

The genome of *P. acidipropionici* comprises a circular chromosome of 3,656,170 base pairs (bp) with 68.8% GC content and a low-copy plasmid of 6,868 bp with 65.4% GC content. The chromosome contains 3,336 protein coding sequences (CDSs) with an average length of 967.5 bp, 53 tRNAs and four 16S-23S-5S rRNA operons, accounting for 88.8% of genomic DNA. Putative functions were assigned to 2,285 (68.5%) of the CDSs, while 556 (16.7%) were classified as conserved hypothetical and 495 (14.8%) had no significant similarity with data in the public databases (e-value >1E-10) (Table 1).

**Table 1:** **General features of *P. acidipropionici *genome**

	**Chromosome**	**pRGO1**
**Length (bp)**	~3,656,170	6,868
**Copy number**	1	~7.4
**Coding content (%)**	88.8	50.5
**G + C content of total genome (%)**	68.8	65.4
**G + C content of coding regions (%)**	69.0	70.8
**G + C content of non-coding regions (%)**	66.9	59.7
**rRNA**	4 X (16S-23S-5S)	0
**Pseudogenes**	32	0
**Genes**	3389	8
**tRNAs**	53	0
**Protein Coding (CDS)**	3336	8
**conserved with assigned function**	2285 (68.5%)	5
**conserved with unknown function**	556 (16.7%)	0
**Nonconserved**	495 (14.8%)	3
**Average CDS length (bp)**	967.5	519.4

Preliminary proteomic analysis of *P. acidipropionici* growing on different carbon sources allowed the identification of 649 (19.5%) of the CDSs (Methods; Additional file 1). The assembled plasmid matched exactly the previously sequenced pRGO1 [13] from *P. acidipropionici* and pLME106 from *P. jensenii*[14]. The number of plasmid copies per cell was estimated to be 7.4 based upon relative read coverage. We identified one new CDS [Genbank:AB007909.1; 1292–1552] in the plasmid coding for an 87 amino acid peptide similar to an InterPro family of proteins putatively involved in plasmid stabilization [InterPro: IPR007712]. This peptide sequence may be useful for the development of *P. acidipropionici* vectors, a fundamental molecular biology tool for the biotechnological use of any organism.

### Comparative analysis

The genome of *P. acidipropionici*, a species that can live in several environments such as soil, rumen and cheese, was compared with the genomes of three closely related species, but with different habitats and ecology: (i) *P. acnes* [GenBank: NC_006085.1], a major inhabitant of human skin and considered to be an opportunistic pathogen that has been associated with acne vulgaris [15]; (ii) *P. freudenreichii subs. shermanii* CIRM-BIA1^T^ [GenBank: FN806773.1] which has known use in cheese manufacture and, more recently, as a probiotic [16]; and (iii) *Microlunatus phosphovorus* [GenBank: NC_015635.1], a species that belongs to the same family (*Propionibacteriaceae*), which is found in soil and has been isolated from activated sludge by its ability to accumulate polyphosphate and polyhydroxyalkanoates (PHA) [17]. The general features of these genomes are summarized in Table 2.

**Table 2:** General features of four species used in comparative analysis

**Organism**	**Genome size (Mb)**	**%GC**	**No. of Proteins**	**No. of rRNA operons**	**No. of tRNAs**
*Propionibacterium acidipropionici* ATCC4875	3.6	68.8	3336	4	53
*Propionibacterium acnes* KPA171202	2.6	60.0	2297	3	45
*Propionibacterium freudenreichii* CIRM-BIA1	2.6	67.3	2375	2	45
*Microlunatus phophovorus* NM-1	5.7	67.3	5338	1	46

**Figure 2:**
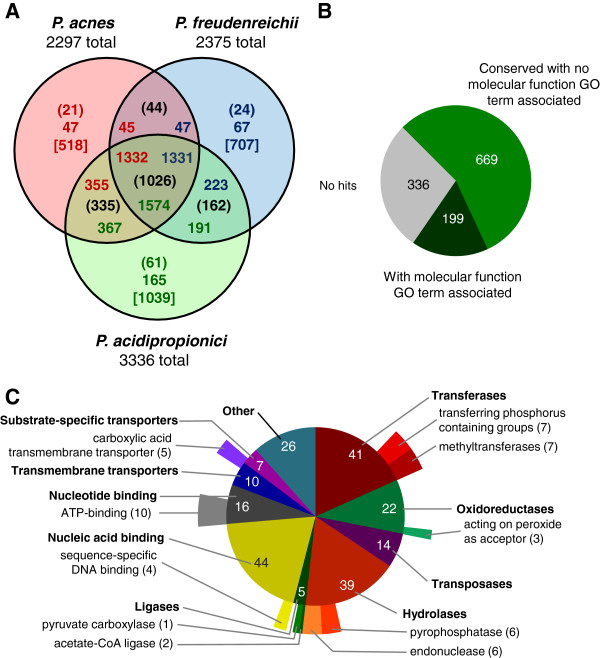
**Comparative protein clustering and GO annotation. A**. Venn diagram showing the number of common and unique protein clusters for three Propionibacteria. The values in parentheses represent the number of protein clusters. The values in square brackets represent the number of single proteins (proteins not in clusters). The number of proteins clustered in each group are also indicated, not enclosed and color-coded by organism: values in green represent *P. acidipropionici* proteins, values in blue represent *P. freudenreichii* proteins and values in red represent *P. acnes* proteins. **B**. Pie chart depicting the result of GO annotation of proteins unique to P. acidipropionici. **C**. Multi-level pie chart detailing GO annotation of proteins unique to *P. acidipropionici*. Inner circle represent level 3 terms. Outer circle represent lower level (more specific) terms.

Clustering of all proteins encoded by these four bacteria resulted in 6469 families. From the total proteins encoded by *P. acidipropionici*, 1009 were clustered into 296 families containing 2 to 54 members. The comparative clustering of proteins from *P. acidipropionici, P. acnes* and *P. freudenreichii* is summarized in Figure 2A, and a four-set Venn diagram including *M. phosphovorus* is presented in an additional figure (Figure S1 in Additional file 2). *P. acidipropionici* shows great expansion in families shared by the three Propionibacteria, most notably in transport proteins, two-component regulatory systems, transcriptional regulators and proteins with oxidoreductase activity (Table 3). Families of transport proteins were compared in detail using the Transporter Classification (TC) system. A total of 469 transport proteins were annotated in *P. acidipropionici* genome and almost half (46.9%) of these proteins were classified as members of the ATP-binding cassette family (ABC). This proportion is greater than in any of the other three bacteria species used in the comparison (Table 4). The complete comparison of transport proteins in these four bacteria species is presented in an additional file (Additional file 3).

**Table 3:** **A selection of paralogous families of *P. acidipropionici *and close related genomes**

**Cluster functional annotation**	**Number of proteins found in:**			
	***P. acidipropionici***	***P. acnes***	***P. freudenreichii***	***M. phosphovorus***
ABC transporters	54	45	34	59
ABC transporters	20	11	1	13
ABC transporters	18	9	1	12
ABC transporters	9	8	4	19
ABC transporters	8	2	4	3
ABC transporters	7	3	2	6
ABC transporters	7	3	2	6
ABC transporters	5	1	2	1
ABC transporters	4	2	1	1
MFS transporters	10	3	10	21
Proton antiporters	3	1	1	2
Anaerobic C4-dicarboxylate transporters	0	2	2	0
Two-component system regulators	24	13	14	48
Two-component system kinases	8	3	4	11
Transcriptional regulators	27	10	4	22
Transcriptional regulators	9	5	5	12
Transcriptional regulators	5	2	1	1
MarR family transcriptional regulators	7	4	4	13
ArsR family transcriptional regulators	3	1	3	20
Oxidoreductases	23	9	8	29
Oxidoreductases	12	8	10	36
Oxidoreductases	12	4	4	21
Oxidoreductases	11	5	7	21
Dehydrogenases	8	2	4	15
Catalases	1	1	1	0
Catalases	1	0	0	1
Glycerol-3-phosphate dehydrogenases	2	1	1	0
Glutathione S-transferases	3	0	1	1
Aminotransferases	8	3	5	8
Aminotransferases	4	1	1	1
Amidotransferases	3	1	1	1
Glycosyl transferases	6	4	3	1
Glycosyl transferases	3	1	0	0
Sugar kinases	4	2	2	0
Polyphospate kinases	1	1	1	1
Polyphospate kinases 2	1	1	1	3
Polyphosphate-dependent glucokinases	1	1	1	2
Lipase esterases	3	1	0	0
Alpha-galactosidase	3	0	0	1
Putative sucrose-6-phosphate hydrolases (invertases)	1	0	0	2
Plasmid maintenance system antidote proteins	3	0	0	0
DNA-binding proteins	2	0	0	11
HNH endonucleases	6	0	0	0
HNH endonucleases	2	0	0	0
Hypothetical proteins	5	1	1	0
Hypothetical proteins	5	0	0	0
Hypothetical proteins	4	3	0	2
Hypothetical proteins	3	0	0	1
Methylases	0	0	2	2
S-layer proteins	0	0	2	0
cAMP factors	0	5	0	0
Adhesion proteins	0	4	0	0
Magnesium chelatase	0	2	0	1
Alpha-L-fucosidases	0	2	0	1
Sialic acid transporters	0	2	0	0
Endoglycoceramidases	0	2	0	0
Adhesion proteins	0	2	0	0
Lysophospholipases	0	2	0	0
Triacylglycerol lipase precursors	0	2	0	0

**Table 4:** **A selection of transporter families of *P. acidipropionici *and close related genomes**

**TC system**	**Description**	**Number of proteins found in:**			
		***P. acidipropionici***	***P. acnes***	***P. freudenreichii***	***M. phosphovorus***
1.C.70	CAMP Factor	0	5	0	0
2.A.1	Major Facilitator Superfamily	38	21	36	68
2.A.1.1	Sugar Porter (SP)	8	5	5	8
2.A.1.3	Drug:H + Antiporter-2 (14 Spanner) (DHA2)	10	3	12	21
2.A.1.6	Metabolite:H + Symporter (MHS)	7	2	4	10
2.A.13	C4-Dicarboxylate Uptake	0	2	2	0
2.A.2	Glycoside-Pentoside-Hexuronide :Cation Symporter	4	1	1	1
2.A.3	Amino Acid-Polyamine-Organocation	12	12	10	6
2.A.47	Divalent Anion:Na+	0	0	2	1
2.A.66	Multidrug/Oligosaccharidyl-lipid/Polysaccharide Flippase	5	1	1	2
3.A.1	ATP-binding Cassette (ABC)	220	137	105	219
3.A.1.1	Carbohydrate Uptake Transporter-1 (CUT1)	57	29	7	40
3.A.1.2	Carbohydrate Uptake Transporter-2 (CUT2)	17	7	4	9
3.A.1.3	Polar Amino Acid Uptake Transporter (PAAT)	19	5	8	11
3.A.1.4	Hydrophobic Amino Acid Uptake Transporter (HAAT)	5	0	5	5
3.A.1.5	Peptide/Opine/Nickel Uptake Transporter (PepT)	28	14	9	29
3.A.1.12	Quaternary Amine Uptake Transporter (QAT)	13	6	7	6
3.A.11	Bacterial Competence-related DNA Transformation Transporter	0	2	2	2
3.B.1	Na + −transporting Carboxylic Acid Decarboxylase	7	3	4	4
4.A.1	PTS Glucose-Glucoside	1	8	1	0
4.A.2	PTS Fructose-Mannitol	2	4	0	0
4.A.3	PTS Lactose-N.N′-Diacetylchitobiose-β-glucoside	0	3	0	0
4.A.4	PTS Glucitol	5	4	0	0
4.A.5	PTS Galactitol	4	2	0	0
4.A.6	PTS Mannose-Fructose-Sorbose	0	0	1	0
4.A.7	PTS L-Ascorbate	0	2	0	0
5.A.3	Prokaryotic Molybdopterin-containing Oxidoreductase	3	6	2	7
8.A.9	rBAT Transport Accessory Protein	4	2	2	6
9.A.10	Iron/Lead Transporter	5	0	2	5
9.A.40	HlyC/CorC Putative Transporters	4	2	2	2

The assignment of Gene Ontology terms to proteins unique to *P. acidipropionici* is depicted in Figures 2B and 2C. Two proteins that distinguish the central fermentative pathway of *P. acidipropionici* from that of *P. acnes* and *P. freudenreichii* have ligase activity and are discussed in the section “Propionic acid fermentation”. Proteins with sequence-specific DNA binding and methyltransferase activities could be involved in DNA repair, gene expression and chromosome replication. These proteins could also compose a restriction modification system serving as defense against foreign DNA. Therefore, the study of these proteins that are unique to *P. acidipropionici* may be important in developing genetic tools suitable for this strain.

**Figure 3:**
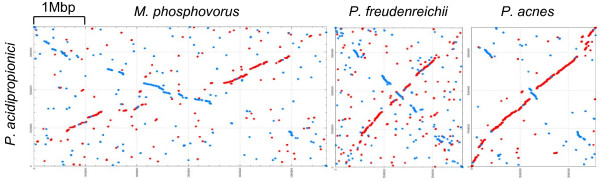
**Syntenic dot plot.** Dot plot alignment between the chromosome of *P. acidipropionici* (vertical) against the chromosomes of *M. phosphovorus*, *P. freudenreichii* and *P. acnes* (horizontal) at protein level. The dnaA gene is located at the beginning of all four sequences.

The number of common genes is consistent with the previously reported phylogeny [18]. *P. acidipropionici* is more closely related to *P. acnes* and colinearity of genes was observed most of the time (Figure 3)*.* However, *P. acnes* has approximately 1,000 genes fewer than *P. acidipropionici*, a fact that could reflect the specialization of this species as an opportunistic pathogen of human skin. It is generally considered that specialization leads to gene loss [19], a process that can be very fast in bacteria. For example, in *Lactobacillus* the transition of strains to nutritionally rich environments leads to metabolic simplification and the loss of several genes [20]. Although *P. freudenreichii* has all the enzymes needed for the de novo biosynthesis of amino acids and vitamins [16], the metabolic simplification premise may indicate that the reduced number of genes found in *P. freudenreichii* in comparison to *P. acidipropionici* is suggestive of the beginning of a specialization process as a consequence of the continuous use of this species in cheese ripening. This reasoning is consistent with the analysis of the *M. phosphovorus* genome, a soil bacterium, with 5,338 annotated genes. Most of the gene clusters that were conserved in the three *Propionibacterium* are also present in *M. phosphovorus* (895 out the 1,026) (Figure 2A; Figure S1 in Additional file 2). *P. acidipropionici*, with 3,336 genes, would seem to hold an intermediary position. The 32 pseudogenes identified in *P. acidipropionici* are disrupted by a single frameshift or point mutation, suggesting that these events are recent and that some genome reduction is underway.

The horizontal transfer of DNA fragments might supply the recipient microorganism with the necessary genetic resources to be able to adapt to new environments; for example, an antibiotic resistance gene and/or a gene that encodes a peptide in a biodegradative pathway [21]. Furthermore, Jain *et al.*[22] described the horizontal transfer of genes as a mechanism to spread genetic diversity across species and showed that horizontal gene transfer occurs between organisms that share similar factors like G/C content, genome size, oxygen tolerance and carbon utilization. Predicted genomics islands account for 3.8% (126) of total *P. acidipropionici* genes. This number is similar to predictions in *P. acnes* and *M. phosphovorus* genomes (3.0% and 4.8%, respectively) but lower than the prediction in the *P. freudenreichii* genome (6.7%).

#### Defense of genome integrity

Analysis of the *P. acidipropionici* genome revealed some defense mechanisms that may allow it to withstand viral and nucleic acid invasion; these include restriction enzymes and the Clustered Regularly Interspaced Short Palindromic Repeats (CRISPRs). CRISPRs can provide the cell with an acquired resistance against bacteriophages and conjugative plasmids, possibly acting as a RNA interference-like mechanism. The spacers between direct repeats are derived from invader sequences and determine the specificity of the system. In addition, the mechanism is composed by CRISPR-related sequences (Cas), which are proteins encoded in the vicinity of CRISPR loci [23]. Some of these proteins show similarity to helicases and repair proteins. Seven CRISPR-associated proteins were annotated in the *P. acidipropionici* genome (Cas1, Cas2, Cas3, Cas5, Cse1, Cse3, Cse4), while only two were identified in the *P. freudenreichii* and *M. phosphovorus* genomes. Moreover, using the CRISPR finder tool (crispr.u-psud.fr/Server/CRISPRfinder.php, [24]), three CRISPR loci were annotated in *P. acidipropionici* genome. The CRISPR1 locus contains 1248 bp and harbors 20 spacer sequences, the CRISPR2 locus contains 2043 bp and harbors 33 spacer sequences and the CRISPR3 locus contains 3689 bp and harbors 60 spacer sequences. The length of direct repeat sequence in all CRISPR loci is 29 bp. The spacers do not show strong similarity to phage and bacteria sequences available in databases from the National Center for Biotechnology Information (NCBI). Since Cas genes and CRISPRs are known to have undergone extensive horizontal transfer and the identified proteins do not share high sequence similarity (were not clustered together), these regions could have been acquired horizontally from different sources, explaining this inequality. No CRISPR-related proteins were found in the *P. acnes* genome.

The most common cause of slow or incomplete bacterial fermentations in the dairy industry is bacteriophage infections that lead to substantial economic loss [25]. To deal with phage diversity, lactic acid bacteria have developed several systems to withstand the infections; one of these is the CRISPR/Cas system. CRISPR/Cas loci have been identified in many lactic acid bacteria, such as *Streptococcus thermophilus*, *Lactobacillus casei*, *Lactobacillus delbrueckii*, *Lactobacillus helveticus* and *Lactobacillus rhamnosus*[26]. The CRISPR/Cas system was found in the genome of *P. acidipropionici*, and could thus be interpreted as another feature of this bacterium that could render it robustness against phage infections in an industrial setting for the production of chemical commodities. Furthermore, the genomic data for the CRISPR/Cas system found in P. acidipropionici will provide information about the defense mechanism of this bacterium and allow the development of studies to improve its resistance to phage infections.

#### Lifestyle and environmental adaptation

Many of the annotated proteins families in *P. acidipropionici* have the potential to render this species flexibility to adapt to different environments (Tables 3 and 4). Among these protein families are the carbohydrate ABC transporters, transcriptional regulators, two-component response regulators, and oxidoreductases; all of these families had greater numbers of members in *P. acidipropionici* when compared with *P.acnes* or *P. freudenreichii*. Moreover, there are more rRNA operons and tRNA genes in *P. acidipropionici* compared with the other three species (Table 2). This feature could potentially confer competitive advantages such as a faster growth rate and quick response to environmental changes [27]. *M. phosphovorus* contains the same set of 45 tRNAs as *P. acnes* and *P. freudenreichii*, plus a selenocysteine tRNA that could be present solely to support formate dehydrogenase expression and is unique to *M. phosphovorus* among the four species that were compared.

Other protein families that appear expanded in *P. acidipropionici* in comparison to the other three species are glutathione-S-transferases (GSTs) and HNH endonucleases. Compared with the single copy of a glutathione S-transferases (GST) coding gene found in *P. freudenreichii* genome, three copies of the gene were found in the *P. acidipropionici* genome (Table 3). In bacteria, this enzyme has been reported to be involved on growth on recalcitrant chemicals and in the degradation of aromatic compounds [28]. HNH endonucleases coding genes were absent from the genome of *P. freudenreichii*, *P. acnes* e *M. phosphovorus*, while eight copies grouped into two clusters were found in *P. acidipropionici* (Table 3). This enzyme is a type of homing endonuclease that could be potentially involved in DNA rearrangements or could act as bacteriocin [29,30]. Taken together, the notable expansion of these two gene families may confer to *P. acidipropionici* robustness in its capacity for growth under adverse conditions and competitiveness against other bacterial species. However, further studies are needed to establish the function of these enzymes in the lifestyle of *P. acidipropionici*.

On the other hand, previously reported probiotic related genes were found only in the *P. freudenreichii* genome (gluconate kinase, S-layer proteins, cell-wall peptidase NlpC/p60, sortase and microcin resistance) [16], thus agreeing with the possible adaptation of the species to the nutritional environment of the gut. Similarly, some protein families that were identified only in *P. acnes* are probably related to pathogenicity and host specialization. Some examples of these families include cAMP factor, endoglycoceramidases, sialic acid transporter, sialidases, and lysophospholipase.

#### Energy reserve and stress resistance

Polyphosphates (polyPs) are linear polymers composed of orthophosphate residues. PolyPs are not only used as energy reserves, but also have been related to stress resistance and adaptation to extreme environments [31]. *P. freudenreichii* ST33 has been reported to accumulate up to 3% of cell dry weight of polyP [32] and *M. phosphovorus* NM-1 has been reported to accumulate up to 11.8% [33]. Falentin *et al.*[16] identified the genes related to polyphosphate metabolism in *P. freudenreichii* CIRM-BIA1; all of these genes were present in the four genomes that were compared in the present study. Similar numbers of the Nudix hydrolases were present in all four organisms, suggesting that they had similar levels of metabolic complexity and adaptability [34]. In addition to the one copy of polyphosphate kinase (PPK) per genome that we found, PPK2 genes were also identified in the four genomes. PPK is an important enzyme in bacterial polyP synthesis, transferring reversibly the terminal phosphate of ATP to polyP. PPK2 differs from PPK by its use of either GTP or ATP, its preference for Mn^2+^ over Mg^2+^ and for being stimulated by polyP [34]. *P. acidipropionici* (PACID_16380), *P. acnes* (GenBank: NC_006085.1; locus: PPA1186) and *P. freudenreichii* (GenBank: FN806773.1; locus PFREUD_12510; annotated as hypothetical) have one PPK2 copy each. *M. phosphovorus* contains three PPK2 copies (Genbank: NC_015635.1; loci: MLP_05750, MLP_23310 and MLP_50300), which may play an important role in the large polyP accumulation in this organism.

### Metabolic reconstruction

**Figure 4:**
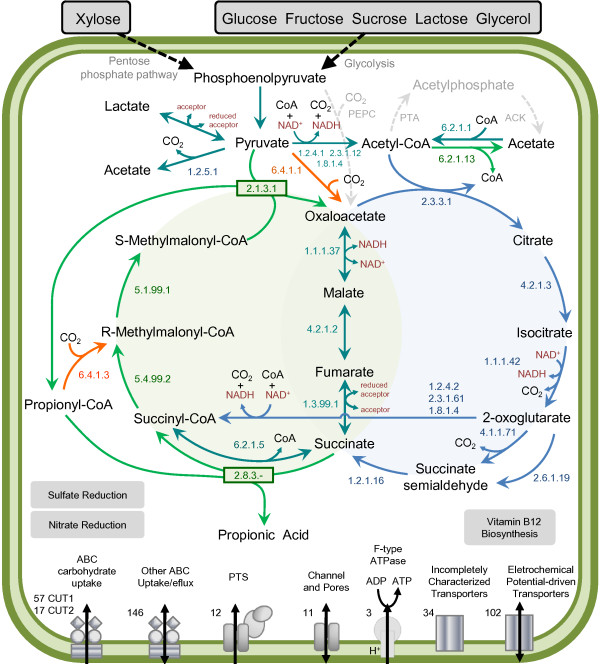
**Overview of *P. acidipropionici *metabolism.** Reactions of fermentative pathway are in green while reactions of respiratory pathway are in blue. Reactions described in literature but absent in the genome are in gray dotted lines. Putative reactions for CO_2_ fixation are in orange. The total number of transporters in each major category are shown.

The predicted metabolic map of *P. acidipropionici* contained 221 pathways and 1,207 enzymatic reactions. The gene annotations and metabolic maps revealed that the complete pathways of glycolysis, gluconeogenesis, pentose-phosphate and all *de novo* amino acid biosynthesis were represented in the *P. acidipropionici* genome. The TCA cycle was also complete and genes corresponding to aerobic and anaerobic respiration pathways were detected. The biosynthetic pathways for most vitamins were found; however, pathways for biotin and pantothenic acid synthesis were notably absent, as has been reported for other species from this genus [16]. Figure 4 summarizes the basic metabolic pathways that were present in *P. acidipropionici*.

#### Substrate utilization

It has been reported that *P. acidipropionici* can use a wide variety of substrates for the heterofermentative production of propionic acid [7]. In agreement with this report, the genome analysis of *P. acidipropionici* has revealed a notable set of transporters (Table 4) and enzymes that could be related to uptake and degradation of numerous substrates, putatively including glucose, fructose, sucrose, lactose, xylose, threalose, mannose, chitobiose and arabinose. The genome comparison showed that two families within ABC superfamily concerned exclusively with carbohydrate uptake are present in greater number in the genome of *P. acidipropionici.* The carbohydrate uptake transporter-1 (CUT1) and −2 (CUT2) families exhibit specificity for oligosaccharides and monosaccharides, respectively. There are two principal amino acid uptake families in the ABC superfamily. The family specific for polar amino acids uptake (PAAT) is more numerous in the *P. acidipropionici* genome. The family concerned with hydrophobic amino acids uptake (HAAT) had the same number of genes identified in all bacteria compared, except in *P. acnes,* where no members were identified [35]. The propanediol utilization operon of *P. freudenreichii,* probably acquired through horizontal transfer [16], is not present in *P. acidipropionici*, *P. acnes* or *M. phosphovorus*.

**Figure 5:**
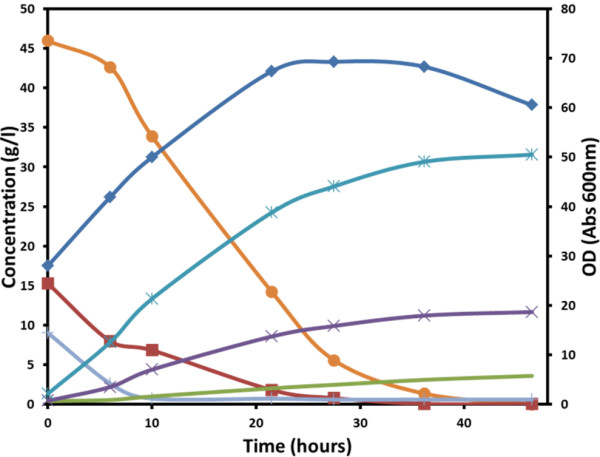
**Preliminary results of *P. acidipropionici *experimental batch fermentation with sugarcane juice.** Biomass A600 (blue diamond); sucrose (orange circle); glucose (red square); fructose (gray line); propionic acid (blue asterisk); acetic acid (purple cross); succinic acid (green line).

*P. acidipropionici* possesses one invertase coding gene (PACID_33010) and could thus potentially use sucrose as a carbon source, while *M. phosphovorus* has two copies of putative invertase coding genes (MLP_06620 and MLP_06630). *P. acnes* and *P. freudenreichii* lack such a gene, and for that reason, the growth of these species in sucrose containing feedstocks like molasses needs pretreatment, with consequent cost increase [36]. This feature enables the development of *P. acidipropionici* fermentations at low cost from renewable feedstocks, such as sugarcane juice readily available in Brazil. Experimental batch fermentations using *P. acidipropionici* with sugarcane juice showed a significant yield and productivity of propionic acid, although significant amounts of acetic and succinic acids still emerged as by-products (Figure 5). In addition, the ability of this bacterium to metabolize the C5 sugar xylose has also been previously reported [37], which makes this species a strong candidate for the metabolization of second generation feedstocks derived from biomass. Industrial ethanol fermentations are commonly contaminated by indigenous yeasts, which many times are less productive than the commercial yeasts used in ethanol fermentation. This causes considerable yield losses and in some cases indigenous yeasts are even able to replace commercial yeasts [38]. Preliminary experiments showed that the production of propionic acid inhibited the growth of indigenous yeasts in sugarcane juice (data not shown). Hence, industrial fermentation to produce propionic acid with *P. acidipropionici* using sugarcane juice could be performed without strict microbial control, potentially reducing production costs.

In summary, we report here the genetic basis for the ability of *P. acidipropionici* to metabolize many carbon sources, confirming previous studies of carbon source utilization by this species. These carbon sources include the readily available sucrose from sugar cane juice [7] and the C5 sugar xylose that is abundant in second generation feedstocks derived from biomass [37]. These results coupled to the preliminary findings suggesting that the propionic acid produced by propionibacteria may help control possible contamination by indiginous yeast species in an industrial setting emphasizes the potential of *P. acidipropionici* as a biological reactor of industrial interest.

#### Catabolic repression and phosphotransferase system

Given the wide range of substrates that *P. acidipropionici* is able to metabolize, we performed a preliminary study to verify the presence of carbon catabolite repression. *P. acidipropionici* was grown in various carbon sources (glucose, fructose, sucrose, lactate and glycerol) with or without addition of the hexose analogous 2-deoxy-glucose (2-DG). 2-deoxyglucose is taken up by cell and phosphorylated into 2-DG 6-phosphate. This molecule cannot be metabolized; however it is able to trigger the glucose repression phenomenon [39]. Therefore, the presence of 2-DG in the cultures led to a clear decrease in the *P. acidipropionici* growth rate, suggesting the presence of a possible mechanism of carbon catabolite repression by glucose (Figure S2 in Additional file 2). The analysis of variance test (ANOVA) indicated that there was a significant difference between the growth rate of *P. acidipropionici* grown in the media containing glucose and in the media containing glucose with 2-DG (ANOVA factorial: F_7.32_ = 8.346; p <0.0001); fructose and fructose with 2-DG (ANOVA factorial: F_7.32_ = 22.363; p < 0.0001); sucrose and sucrose with 2-DG (ANOVA factorial: F_7.32_ = 35.70; p < 0.0001); lactate and lactate with 2-DG (ANOVA factorial: F_7.32_ = 24.53; p < 0.0001) and glycerol and glycerol with 2-DG (ANOVA factorial: F_7.32_ = 11.46; p < 0.0001).

The molecular basis of this phenomenon is generally related to a multiprotein phosphorelay system, the phosphotransferase system (PTS). At least three enzymes, enzyme I (EI), histidine protein (HPr) and enzyme II (EII), that are responsible for carbohydrate transporting, phosphorylation and triggering the catabolic repression respectively, are present in the PTS [40]. Twenty-three genes coding for general proteins of the PTS were identified in the *P. acidipropionici* genome (Additional file 4). Moreover, two transcriptional factors probably involved in this system (CRP – cyclic AMP receptor factor and ccpA – catabolite control protein A) and 1 PTS-regulatory domains (PRDs), which could act as antitermination proteins or as transcriptional activators, were found in the genome of *P. acidipropionici*. In addition, carbon catabolite repression can regulate the expression of virulence factors in many pathogenic bacteria [40], and this could explain the great number of PTS related genes (31 genes) found in the *P. acnes* genome. On the other hand, *P. freudenreichii* has only 5 genes related to the PTS. These observations could suggest that *P. acidipropionici* has the potential ability for selective carbon source utilization and genome plasticity to adapt to different environments, but further studies would be necessary in order to confirm this hypothesis.

#### Aerobic metabolism

*P. acidipropionici* is a facultative anaerobic microorganism. In practical terms, the cells need anaerobic conditions to grow on solid media; however, they can grow in liquid media with oxygen added (data not shown) [41]. Inspection of its genome showed that all the genes related to aerobic respiration and oxidative stress are present. *P. acidipropionici* had two putative catalase genes while *P. acnes* and *P. freudenreichii* contained only one copy. Thus, the susceptibility of *P. acidipropionici* to oxygen could be related to deficient expression of the oxidative defense genes or to a redox imbalance. These preliminary data are encouraging in suggesting oxygen resistance in this species. However, further studies on the oxygen resistance of *P. acidipropionici* will be needed in order to establish non-strict anaerobic conditions for growth in an industrial setting, where the maintenance of a strict anaerobic atmosphere would be too costly.

#### Electron transport chain

The electron transport chains of prokaryotes vary widely, having multiple terminal oxidases. This variation allows bacteria to adapt their respiratory systems to different environmental growth conditions [42] by choosing the composition of enzymes that will achieve: (i) the highest possible coupling efficiency (H^+^/e^-^ ratio); (ii) the rapid removal of excess reducing equivalents such as NADH/NADPH; and (iii) the regulation of intracellular oxygen concentration [43].

Cytochrome bd (PACID_05290 and PACID_05280) is present in all four organisms that were compared. In *E. coli*, this oxidase has a high affinity for oxygen but it does not pump protons being preferred in low-oxygen conditions because the respiratory chain is then still able to provide energy, even in low amounts [44]. Although the cytochrome *bd* complex does not pump protons in *E.coli*, there is a net proton transfer (1 H^+^/e^-^) by this oxidase due to quinol oxidation and oxygen reduction on the periplasmic and the cytoplasmic sides of membrane, respectively [44-46].

On the other hand, cytochrome c oxidase (CcO) reduces molecular oxygen to water coupled with the pumping of four protons across the membrane, generating more energy [47]. The four subunits of CcO (PACID_12210-12230 and PACID_12290), the cytochrome C reductase (PACID_12260, PACID_12280 and PACID_12290) and cytochrome c biogenesis and assembly proteins were found in *P. acidipropionici, P. acnes* and *M. phosphovorus,* suggesting a greater flexibility of these strains to adapt to different oxygen conditions in comparison to *P. freudenreichii,* which lacks these genes. However, *P. acidipropionici* showed a frameshift in CcO subunit I (Figure S3 in Additional file 2). Because the CcO subunit I is a strongly conserved protein and the *P. acidipropionici* version have lost most of its domain identity and its copper-binding site, this oxidase is probably not functional. The alignment of the 454 and Illumina reads against the frameshift region can be viewed in the additional figure S4 (Additional file 2).

A recent transcriptome work using *P. acnes*[48] detected expression of the whole respiratory chain under anaerobic growth conditions, including NADH dehydrogenase/complex I (NDH-1) and succinate dehydrogenase/fumarate reductase (SdhABC). Wackwitz and colleagues [49] showed that NDH-1 is stimulated under fumarate-dependent respiration in *E. coli*. Brzuszkiewicz and colleagues [48] hypothesized a scenario of *P. acnes* metabolism with ATP generation via F_o_F_1_ ATP synthase and proton translocation by NDH-1 and SdhABC, with the former feeding reducing equivalents to the respiratory chain, and the latter using fumarate as terminal electron acceptor. These proteins were also detected in our proteome analysis of anaerobic fermentation samples, indicating a similar behavior in *P. acidipropionici,* capable to use different electron acceptors (NDH-1: PACID_26280-26410; F_o_F_1_ ATP synthase: PACID_19340-19410; SdhABC: PACID_21310-21330). *P. acidipropionici* has two SdhABC/FrdABC gene clusters, SdhABC (PACID_21310-21330) and SdhA2B2C2 (PACID_18400-18420). Only SdhABC was detected in the anaerobic fermentation samples and hence could be acting as fumarate reductase, while SdhA2B2C2 could be used in the oxidation of succinate to fumarate. The membrane anchor subunits SdhC and SdhC2 have five transmembrane helices each and predicted molecular weight of 25 and 28 kDa respectively, characteristic for Sdh/Frd proteins lacking Subunit D. When the cluster contains a SdhD/FrdD subunit, the SdhC/FrdC proteins are distinctly smaller (13 to 18 kDa) and contain only three transmembrane helices each [50]. Given its importance in propionibacteria metabolism, a better understanding of electron transport chain can help design engineered strains with fine-tuned redox balance.

#### Nitrate and sulfate reduction

Kaspar (1982) [51] demonstrated that strains of *P. acidipropionici*, *P. freudenreichii, P. jensenii, P. shermanii* and *P. thoenii* can reduce nitrate to nitrite and further to nitrous oxide. This ability was later shown to vary from strain to strain, being strongly affected by environmental factors [52]. *P. acidipropionici* ATCC 4875 seems to be able to use nitrate as an electron acceptor, since all the genes of the respiratory nitrate reduction to nitrous oxide were all found: respiratory nitrate reductase (EC:1.7.99.4; PACID_02700-02730), nitrite reductase (cytochrome) (EC:1.7.2.1; PACID_02330) and nitric-oxide reductase (cytochrome c) (EC:1.7.2.5; PACID_31710 and PACID_27170). These genes are also present in *P. acnes* and *M. phosphovorus*, while *P. freudenreichii* subsp. *shermanii* cannot reduce nitrate due to lack of a functional nitrate reductase [16]. Also, *P. acidipropionici* could reduce the nitrite to ammonium through the enzyme nitrite/sulfite reductase (EC:1.7.7.1; PACID_27260) and incorporate the ammonium formed into amino acids. This gene is also present in *P. freudenreichii* and *M. phosphovorus*, but not in *P. acnes.* Additionally, genes coding for an assimilatory nitrite reductase (EC:1.7.1.4; PACID_33080 and PACID_33090) are found in *P. acidipropionici* and *M. phosphovorus*. However, the enzyme of the former is nonfunctional due to a frameshift in the large subunit.

The sulfur assimilation pathway also varies among propionibacteria, with species utilizing from the most oxidized sources (sulfate) to the most reduced ones (sulfide) [53]. An adenylylsulfate kinase (EC:2.7.1.25) was only found in *M. phosphovorus.* The coding genes for nitrite/sulfite reductase (EC:1.7.7.1; PACID_27260), sulfate adenylyltransferase (EC:2.7.7.4; PACID_01720 and PACID_01730) and phosphoadenylyl-sulfate reductase (EC:1.8.4.8; PACID_01710) coding genes were found in the genomes of *P. acidipropionici, P. freudenreichii* and *M. phosphovorus,* but not in *P. acnes.* However, it was not possible to identify the complete pathway of sulfate reduction in *P. acidipropionici,* thus requiring more studies.

*P. freudenreichii* and *P. acnes* have genes coding for all three subunits of anaerobic dimethyl sulfoxide (DMSO) reductase, allowing these bacteria to use DMSO and other highly oxidized substrates as electron acceptors.

#### Vitamin B12 biosynthesis

Cobalamin (vitamin B12) is one of the most structurally complex nonpolymeric biomolecule described and is an essential cofactor for several important enzymes like methylmalonyl-CoA mutase [48]. The complexity of its synthesis makes a chemical production too challenging and expensive. Hence, the industrial production of this vitamin is currently performed by biosynthetic fermentation processes using two organisms: *Propionibacterium freudenreichii (P. shermanii)* and *Pseudomonas denitrificans*. Since some *Propionibacterium* species do not produce either endo- or exotoxins, they are preferred for the production of food additives or medicines. *Pseudomonas* produce Vitamin B12 by the aerobic pathway, whereas *Propionibacteria* use the anaerobic one, which differs from the first in that the cobalt ion is inserted much earlier, at the precorrin-2 intermediate. The oxidation step also differs as it cannot use O_2_ and generates a 6- rather than a 5-membered lactone [54].

*M. phosphovorus* uses the aerobic pathway and the characteristic oxygen-requiring C-20 hydroxylase CobG and the distinct cobalt insertion complex (cobNST) were identified [Genbank: AP012204.1]. The organization of B12 biosynthesis genes of *P. acidipropionici* is similar to that of *P. acnes*, with genes grouped into two clusters (Figure S5 in Additional file 2), while *P. freudenreichii* has its genes grouped into four gene clusters. The small cluster (PACID_28120-28220) harbors genes responsible for providing aminolaevulinic acid and converting it to uroporphyrinogen III, as well as the interconversion of uroporphyrinogen III into haem. The large cluster (PACID_08770-09000) harbors genes responsible for cobalt transport and adenosylcobalamin synthesis from uroporphyrinogen III. Some genes coding for transport proteins are only present in the cluster from *P. acnes.* Fused genes also differ between the three genomes. While cbiEGH appears in the three Propionibacteria, cobT, cobU and bluB are organized differently. *P. acnes* large cluster harbors cobT and cobU genes fused into a single protein (cobTU), while *P. acidipropionici* large cluster contains two separate adjacent genes (cobT:PACID_08960 and cobU:PACID_08970). *P. freudenreichii* have cobU as a separate gene inside one cluster, and cobT fused to the bluB gene and located elsewhere in its genome. This fused gene, cobT/bluB, is also present in the *P. acnes* genome, but a bluB homolog was not found in *P. acidipropionici* genome. BluB, first identified in *Rhodobacter capsulatus,* was shown to be required for the conversion of cobinamide to cobalamin in various organisms, being responsible for the oxygen-dependent synthesis of dimethylbenzimidazole from reduced flavin mononucleotide (FMNH_2_) [55]. Since *P. acidipropionici* does not possess this gene, another unidentified enzyme may catalyze this reaction.

#### Propionic acid fermentation

As expected, the dicarboxylic acid pathway, known as Wood Werkman cycle, was identified in the *P. acidipropionici* genome (Figure 4). Many bacteria of different genera as *Rhodospirillum*, *Mycobacterium*, *Rhizobium*, *Micrococcus* and *Propionibacterium* have the mechanisms required for propionic acid fermentation metabolism [56]. Nonetheless, it is only in *Propionibacterium* that this is the main pathway for energy generation, resulting in high propionic acid production. Propionic acid production is a cyclic process, in which propionate formation is related to the oxidation of pyruvate to acetate and to reduction of fumarate to succinate. We identified thirteen genes in the *P. acidipropionici* genome that could encode the enzymes involved in propionate production from pyruvate: methylmalonyl-CoA carboxyltransferase (EC:2.1.3.1; PACID_07970-08000), malate dehydrogenase (EC:1.1.1.37; PACID_24560), fumarate hydratase (EC:4.2.1.2; PACID_33440), succinate dehydrogenase (EC:1.3.99.1; PACID_21310-21330), propionyl-CoA:succinate CoA-transferase (EC:2.8.3.-; PACID_06950), methylmalonyl-CoA epimerase (EC:5.1.99.1; PACID_19260) and methylmalonyl-CoA mutase (EC: 5.4.99.2; PACID_11200 and PACID_11210).

Importantly, two main divergences from the literature were found. The phosphoenolpyruvate carboxytransphosphorylase (PEPC) that would lead to the formation of oxalacetate from CO_2_ and phosphoenolpyruvate is not present in *P. acidipropionici, P. acnes*, *P. freudenreichii* or *M. phosphovorus* genomes. In addition, the acetate dissimilation pathway differs from previous reports [7,57]. *P. acidipropionici* does not contain the phosphate acetyltransferase (PTA) and acetate kinase (ACK) genes; therefore, the two step PTA-ACK pathway cannot be the one that is used for acetate assimilation or dissimilation as was thought previously. Instead, this bacteria seems to dissimilate acetate using an ADP-forming acetyl-CoA synthetase (ADP-ACS, EC:6.2.1.13 PACID_02150), an enzyme that is distinct from the broadly distributed AMP-ACS and which is instead related to the ADP-forming succinyl-CoA synthetase complex (SCSC) [58]. Unlike PTA and ACK, ADP-ACS converts acetyl-CoA, inorganic phosphate and ADP into acetate, ATP and CoA in one step [59]. The protein encoded by ADP-ACS gene was found in all proteome samples of *P. acidipropionici*, where acetate production was also observed. The PTA and ACK genes were identified in the genomes of *P. freudenreichii* and *M. phosphovorus* but not in the genomes of *P. acidipropionici* and *P. acnes*. An ADP-ACS gene was only identified in *P. acidipropionici* and related genes from the SCSC (α and β subunits) were also found in all the compared organisms except *P. freudenreichii*. The acetate assimilation is probably performed by AMP-forming ACS enzymes (6.2.1.1; PACID_13940 and PACID_13950). Although reversible in vitro, the reaction carried out by these enzymes is irreversible in vivo because of the presence of intracellular pyrophosphatases. In *E. coli,* this high affinity pathway scavenges small amounts of environmental acetate, while PTA-ACK pathway works only with large concentrations of the substrate [60]. In this way, *P. acidipropionici* resembles some halophilic archaea which do not have the PTA-ACK pathway and use AMP-ACS and ADP-ACS enzymes to assimilate and dissimilate acetate, respectively.

#### n-Propanol production

In addition to propionic, succinic and acetic acid, it has been reported that n-propanol might be produced under specific conditions; for example, in the fermentation of *P. acidipropionici* using glycerol as the carbon source [61]. In this case, n-propanol production by *P. acidipropionici* could have resulted by a necessity to balance the intracellular redox potential, since glycerol is more reduced when compared to glucose [62]. The genome analysis identified a possible metabolic pathway for n-propanol production: propionyl-CoA could be converted to propionaldehyde and then to n-propanol by oxidation-reduction reactions catalyzed by the aldehyde and alcohol dehydrogenase enzymes. Genes coding for aldehyde dehydrogenases that could act on propionyl-CoA are PACID_33630, PACID_02980 and PACID_24580. The genome of *P. acidipropionici* contains many alcohol dehydrogenase enzymes, and one or more could be responsible for producing propanol, including PACID_02970, PACID_04160, PACID_05040, PACID_05370, PACID_05800, PACID_06750, PACID_07130, PACID_08230, PACID_11040, PACID_11390, PACID_14120, PACID_16510, PACID_30120, PACID_30260, PACID_30590, PACID_30730, PACID_30890, PACID_31640, PACID_32980, PACID_33650 and PACID_33970.

Indeed, to change the fermentation pattern of *P. acidipropionici* towards higher yields of reduced compounds like propionic acid and n-propanol, it would be necessary to modify the NAD(P)H:NAD(P) cofactor ratio. The influence of the NAD(P)H:NAD(P) ratio in metabolic pathways has been demonstrated, for example, by using substrates with different oxidation states [61] or by supplementing anaerobic growth with electron acceptors, such as nitrate and fumarate [62]. Some studies have also report that the cofactor ratio can be varied through NADH regeneration by the NAD-dependent formate dehydrogenase found in some species of yeast and bacteria. This enzyme catalyzes the oxidation of formate to CO_2_ and the reduction of NAD to NADH [63]. Moreover, the intracellular redox potential could be modified in the presence of a low-potential electron mediator, which transfers electrons between a working electrode and a bacterial cell. Fermentation studies carried out with different strains of *Propionibacterium* in a three-electrode amperometric culture system showed that high yields of the reduced compounds could be produced from oxidized substrates, such as glucose and lactate [64,65]. n-propanol production was also observed in bioelectrical reactors with *P. acidipropionici* ATCC4875 fermenting sucrose in different mediator concentrations [66].

#### CO_2_ fixation

Heterotrophic CO_2_ fixation was reported for the first time in *P. pentosaceum*, later renamed as *P. acidipropionici*[67]. Wood and Leaver [68] tested the fermentation of 3, 4, 5 and 6 carbon compounds by this bacterium and found that the best CO_2_ assimilation occurred when cells were grown on a medium containing glycerol, yeast extract, phosphate and vitamin B. In the same study, they also reported the inhibition of CO_2_ fixation by NaF (Sodium Fluoride). In 1961, Siu *et al.*[69] reported that the PEPC enzyme obtained from sonic extracts of *P. shermanii* was able to catalyze the formation of oxaloacetate from CO_2_ and phosphoenolpyruvate. PEPC is widespread in plants, algae, cyanobacteria, bacteria and protozoa and has a highly conserved domain; the bacterial proteins have approximately 870 residues [70]. However, similarity searches did not identify a homolog of PEPC in the genomes of *P. acidipropionici P. acnes*, *P. freudenreichii* and *M. phosphovorus*.

**Figure 6:**
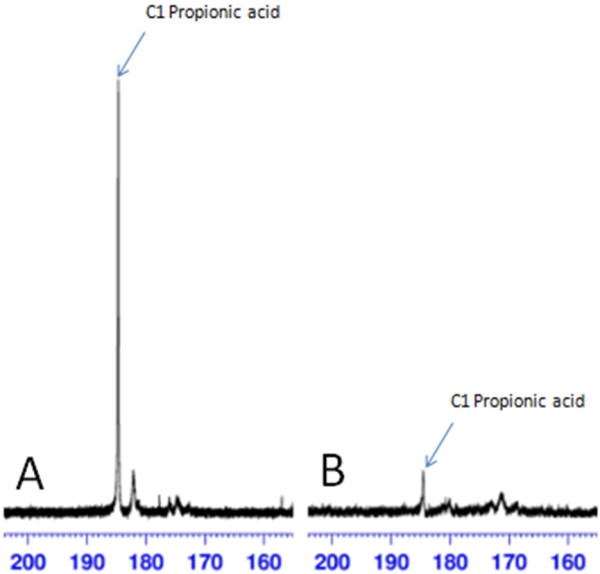
***P. acidipropionici *culture under CO**_**2**_**atmosphere.A**, Culture under ^13^CO2 (labeled) and **B**, Culture with CO_2_ (unlabeled).

To investigate the CO_2_ assimilation by *P. acidipropionici* we performed fermentation assays using glycerol as the substrate in the presence of ^13^CO_2_. The NMR results (Figure 6) revealed an increase in the carbonyl group peak of propionic acid in relation to the control, indicating that a certain amount of isotope ^13^C was fixed by the CO_2_ assimilation pathway. Hence, we concluded that *P. acidipropionici* can indeed fix CO_2_; however, the enzyme responsible for this reaction may not be PEPC. An inspection of the *P. acidipropionici* genome led to the identification of a gene encoding pyruvate carboxylase (EC 6.4.1.1, PACID_00400), an enzyme that catalyzes the following reaction:

(none) $$\;\begin{array}{ccc} \text{Pyruvate} & \;+ & \text{ATP}+{{\text{HCO}}_{3}}^{-}+{\text{H}}^{+}\to \text{oxalacetate}\\ & \;+ & \text{ADP}+\text{phosphate} \end{array}$$

A pyruvate carboxylase was also identified in the *M. phosphovorus* genome (MLP_46180) but not in the *P. acnes* and *P. freudenreichii* genomes. CO_2_ assimilation by *P. acidipropionici* occurs mainly when glycerol is used as the carbon source [68]. Therefore, this pathway could be related to the necessity to balance the intracellular redox potential when the glycerol reaches a high reduction potential. Moreover, in the presence of glucose as carbon source the gene for carbon dioxide fixation could be repressed due to a carbon catabolite repression mechanism [71]. The proteome experiments led to the identification of this protein in all samples obtained from *P. acidipropionici* cells growing in glycerol, but not from cells grown in glucose (data not shown). Although this result supports our hypothesis, further experiments should be performed to elucidate the CO_2_ assimilatory pathway and its regulation.

The pyruvate carboxylase is member of the biotin-dependent carboxylase family. Members of this family are post-transcriptionally biotinylated by a biotin protein ligase, usually present at one copy in prokaryotes and able to biotinylate different carboxylases. Prokaryotes have either one monofunctional protein that only performs the biotinylation or a bifunctional protein containing an N-terminus regulatory domain that participates in the transcriptional control of genes involved in biotin biosynthesis [72]. The four bacteria compared seem to have one copy each of the monofunctional protein, since the typical N-terminal biotin operon repressor domain [TIGRFAMs: TIGR00122] was not found in these proteins.

The heterotrophic CO_2_ fixation emphasizes the potential use of *P. acidipropionici* in industrial applications, once it enables the industrial propionic acid production from low feedstock amounts and fermentation released CO_2_.

## Conclusions

The genome-scale information that we have obtained combined with already developed physiological, genetic and metabolic approaches will increase our knowledge of *P. acidipropionici* and may help establish strategies that will increase and optimize its use in industrial processes. The experimental results and genome annotations have revealed some *P. acidipropionici* features that may explain the plasticity and hardiness of this specie. *P. acidipropionici* has about 1,000 genes more than *P. acnes* and *P. freudenreichii*, suggesting less specialization and a more flexible metabolism. Genes for the defense of genome integrity by the CRISPR/Cas system, polyphosphate accumulation, and the large amount of rRNA and carbohydrate ABC transporters that were identified might also render some competitive advantages and allow the bacterium to adapt in different environments. In addition, the high acid tolerance, wide range of substrates metabolized, hardiness and microaerophilic life style suggest that *P. acidipropionici* is a microorganism that has a potential use in the industrial fermentation of commodities, such as propionic acid. Moreover the antifungal property of propionic acid will help avoid the contamination of the process by indigenous yeast, the main source of contamination in sugarcane juice [38]. Propionic acid fermentation from sugarcane juice, therefore, could be performed without strict microbial control and, consequently, in a cost-effective manner.

Worldwide interest in chemical compounds produced by biological reactors has increased considerably, especially in the petrochemical industry that has as a main objective, the low cost production of compounds that could be fixed in high durable materials. In this context, propionic acid fermentation by *P. acidipropionici* shows a great potential to satisfy the world demand for this commodity.

## Methods

### Bacterial strain

The strain, *Propionibacterium acidipropionici* ATCC 4875™, was acquired from the Global Bioresource Center (ATCC). The genomic DNA was isolated from exponential-phase cultures using previously described methods [73].

### Sequencing and assembly

The genome of *P. acidipropionici* was sequenced by the whole genome shotgun approach using the 454 Life Sciences platform [74] generating single-end 400 bp reads (18.0-fold coverage) and the Illumina/Solexa platform [75] generating mate-pairs of 2 × 75 bp from 3500 bp fragments (108.6-fold coverage). The Illumina reads were submitted to the k-mer based correction tool of SOAPdenovo [76] and trimmed at various lengths prior to assembly. The best results were obtained with reads trimmed to 62 bp. The 454 reads were assembled using GS *de novo* Assembler [NEWBLER version 2.0.00; Roche], resulting in 1,117 contigs and a N50 of 5,274 bp. A hybrid assembly using Velvet version 1.0.04 [77] was then performed to combine the 454 contigs and the Illumina mate-pair reads. Using the VelvetOptimiser tool with the k-mer parameter varying from 35 to 65 bp, the k-mer and coverage cutoff parameters of the final assembly were optimized to 49 bp and 54.3, respectively. The eight resulting chromosome scaffolds were ordered using the *P. acnes* genome as a reference with help of PROmer alignment [78] and dot plot graph tools. The copy number of plasmid per cell was estimated based on the coverage ratio between the corresponding contig and the chromosome contigs. Remaining gaps were closed with just 25 rounds of polymerase chain reactions (PCR) followed by Sanger sequencing.

### Genome annotation

*P. acidipropionici* putative protein-coding sequences were predicted using Glimmer version 3.02 [79]. The training set was composed by *P. acidipropionici* long ORFs and ORFs with complete alignment against *P. acnes* genome found using the Exonerate program [80]. A position weight matrix (PWM) representing ribosome binding sites was constructed by running ELPH [81] on the 25 bp regions upstream of the predicted start sites. The PWM and start codon distribution were supplied to Glimmer to help improve the accuracy of start site predictions. The predicted gene models were manually verified using BLASTX searches against the protein databases Uniprot [82] and NCBI-nr [83]. Automated annotation was performed by AutoFACT [84] which applies similarity searches against UniRef [85], KEGG [86], NCBI-nr [83], Pfam, Smart and COG databases [87], Gene Ontology (GO) terms were assigned using Blast2GO program [88]. The annotations were complemented by searches for conserved domains using RPS-BLAST against the CDD database [87] and important genes were manually verified. Transfer RNA genes were annotated using tRNAscan-SE version 1.23 [89] with the default parameters for bacteria. Ribosomal RNA genes where annotated with RNAmmer version 1.2 [90]. Pseudogenes were identified by direct comparison with homologs and subsequent verification against original sequencing data. Transport protein coding genes were annotated using similarity searches against the Transporter Classification Database (TCDB) [91].

### Comparative analysis

Similarity-based clustering of translated protein sequences was carried out using the Blastclust program [92], with minimum identity ranging from 25–40% and length coverage ranging from 50–80%. The final set of protein families was generated with identity and coverage parameters of 30% and 60%, respectively. Venn diagrams (Figure 2 and Additional file 2: Figure S1) were constructed based on the protein clustering to indicate: (i) protein clusters, text in parenthesis; (ii) proteins not in clusters, text in brackets; (iii) proteins clustered in each group, text not enclosed; (iv) In Figure 2, proteins clustered in each group and separated by organism, color-coded text not enclosed. A detailed comparison of transporter proteins was performed using the TC system [91]. Putative genomic islands were predicted using IslandViewer [93].

### Metabolic maps

The metabolic maps of *P. acidipropionici* were predicted using Pathway Tools version 13.0 [94] and searches against the KEGG database. Metabolic pathways of interest were manually annotated to eliminate false positives and to search for missing enzymes and reactions.

### Culture conditions and media

*P. acidipropionici* was grown in a synthetic medium containing (per liter): 1 g KH2PO4, 2 g (NH4)2HPO4, 5 mg FeSO4·7H2O, 10 mg MgSO4·7H2O, 2.5 mg MnSO4·H2O, 10 mg CaCl2·6H2O, 10 mg CoCl2·6H2O, 5 g yeast extract (HiMedia Laboratories, Mumbai, India), 5 g peptone S (Acumedia Manufacturers Inc, Michigan), and a carbon source (glucose, fructose, sucrose, lactose or glycerol). The medium (without a carbon source) was autoclaved at 121°C and 15 psig for 20 min. The carbon source was autoclaved separately to avoid undesirable reactions, and mixed with the medium aseptically.

### Sugarcane juice batch fermentation

Batch fermentation was performed using sugarcane juice as the carbon source. The synthetic medium described above was supplemented with the juice and the culture mixture was then autoclaved inside the culture vessel. Sugarcane juice free-cell batch fermentation was conducted in a Biostat B. 2.5 L fermenter vessel (B. Braun Biotech International, Melsungen, Germany) containing 2 L of culture medium. The temperature was set at 30°C and the pH was maintained at 6.5 by automatic addition of 4 M NaOH, with 100 rpm agitation. Anaerobiosis was maintained by N_2_ sparing through the culture medium before fermentation began and after each sampling. The bioreactor was inoculated with 200 mL of *P. acidipropionici* cells in the exponential phase (OD ~ 2 to 3); the cells had been grown in PP-test tubes at 30°C. Samples were collected every 24 hours. After measuring the optical density (OD), the remaining volume of the sample was centrifuged at 10,000 g for 6 min. The supernatant was stored at −20°C until high-performance liquid chromatography (HPLC) analysis.

### Analytical methods

Cell biomass was calculated by measuring the absorbance at 600 nm in a ULTROSPEC 2000 spectrophotometer UV/visible (Pharmacia Biotech) after appropriate dilution in water. For HPLC-RI analysis, the samples were filtered through a 0.2 μm filter (Millipore). Propionic, succinic and acetic acids and sugars were determined by HPLC (Waters 600 Chromatograph), using an ion exclusion column Aminex HPX-87 H (Bio-Rad). The operation temperature was 35°C and 0.16 M H_2_SO_4_ was used as the mobile phase at a flow rate of 0.6 mL/min.

### Catabolic repression assays

The bacteria were cultured in media (described above) containing 6 mM 2-deoxy-glucose (2-DG) and one of the following carbon sources: glucose, fructose, sucrose, lactose or glycerol at a concentration of 5 g/L. The cultures were incubated, without agitation or pH regulation, at 30°C for 9 days. Bacterial growth in the different carbon sources was analyzed by OD_600_ determination in a ULTROSPEC 2000 spectrophotometer UV/visible (Pharmacia Biotech) after appropriate dilution in water. The results were tested by analysis of variance (ANOVA).

### CO_2_ assimilation assays

The cultures were carried out in 9 mL sterile serum tubes with rubber caps, containing 6 mL of culture medium with 10 g/L of glycerol as the carbon source and 3 mL of headspace for CO_2_ or N_2_ injection. The culture medium was injected with a sterile syringe. The tubes were inoculated with 1 mL of *P. acidipropionici* cells in the exponential growth phase (OD_600_ ~ 2 to 3); the cells had been grown in polypropylene test tubes at 30°C. Immediately, the headspace was saturated with the respective gas at room temperature. The headspace gas was mixed with the medium by vigorous agitation before starting the culture. The cultivation tests were performed with carbon-^13^C dioxide (99 atom % ^13^C, 3 atom % ^18^C, Sigma-Aldrich) so that the nuclear magnetic resonance (NMR) determination of CO_2_ absorption by analysis of fermentation products could be performed. As controls, other test tubes were inoculated following the same procedure but using either the standard CO_2_ or N_2_ gases. Each cultivation test was performed in triplicate. The tubes were incubated without agitation or pH regulation at 30°C for 3 days. Afterwards, the tubes were centrifuged at 10,000 g for 6 min, and the supernatant was lyophilized and resuspended in D_2_O for NMR analysis.

### NMR spectroscopy

The ^13^C NMR spectra at 500 MHz (11.740 Tesla) were obtained in a Varian INOVA spectrometer (Varian) with a 5 mm diameter probe. The spectrum consisted of 5000 pulses, and the span between pulses was 1.5 s.

### Proteome experiments

The bacterial cells were grown in two different media, one containing 60 g/L of glucose and the other 60 g/L of glycerol both at 37°C, 200 rpm and keeping the pH at 6.5. The fermentation was performed in a 2 L batch fermenter (Labfors, Infors, AG, Switzerland). The glucose culture was grown for 168 h (OD_600_ = 17) and the glycerol culture was grown for 216 h (OD_600_ = 12). Samples of the glucose fermentation were taken after 11 (OD_600_ = 0,6), 24 (OD_600_ = 8,8), 72 (OD_600_ = 22,1) and 120 (OD_600_ = 22,7) hours and the glycerol fermentation was sampled after 11 (OD_600_ = 1,5), 72 (OD_600_ = 6,7), 144 (OD_600_ = 19,3) and 216 (OD_600_ = 12,1) hours. The fermentation samples for proteome analysis (UPLC-ESI-Q/TOF) were treated according to the protocol for mammalian cultured cells described previously [95], except that the sonication was done 3 times for 50 s with a 3 min rest in between using output 20%. The samples were fractionated by strong cation exchange chromatography before injection in the UPLC-ESI-Q/TOF system.

### Nucleotide sequence accession numbers

The sequence of *P. acidipropionici* strain ATCC 4875 genome is available in GenBank under the accession number CP003493.

## Abbreviations

PLA: Polylactic acid; bp: Base pairs; CDSs: Protein coding sequences; PHA: Polyhydroxyalkanoates; TC: Transporter Classification; ABC: ATP-binding cassete; CRISPRs: Clustered regularly interspaced short palindromic repeats; NCBI: National Center for Biotechnology Information; GST: Glutathione S-transferase; PolyPs: Polyphosphates; PPK: Polyphosphate kinase; CUT1: Carbohydrate uptake transporter-1; CUT2: Carbohydrate uptake transporter-2; PAAT: Polar amino acids uptake transporter; HAAT: Hydrophobic amino acids uptake transporter; 2-DG: 2-deoxy-glucose; ANOVA: Analysis of variance; PTS: Phosphotransferase system; EI: Enzyme I; HPr: Histidine protein; EII: Enzyme II; CcO: Cytochrome c oxidase; NDH-1: NADH dehydrogenase/complex I; SdhABC: Succinate dehydrogenase/fumarate reductase; DMSO: Dimethyl sulfoxide; PEPC: Phosphoenolpyruvate carboxytransphosphorylase; PTA: Phosphate acetyltransferase; ACK: Acetate kinase; ADP-ACS: ADP- forming acetyl-CoA synthetase; SCSC: ADP-forming succinyl-CoA synthetase complex; ATCC: The Global Bioresource Center; GO: Gene Ontology; TCDB: Transporter Classification Database; OD: Optical density; HPLC: High-performance liquid chromatography; NMR: Nuclear magnetic resonance.

## Competing interests

The authors declare that they have no competing interests.

## Authors’ contributions

LPP conceived and designed the experiments, carried out the bioinformatics analysis, analyzed the genome data and wrote the manuscript; MCBG conceived and designed the experiments, carried out the molecular experiments, analyzed the genome data and wrote the manuscript; VLQ performed the proteome experiment; LLC performed the batch fermentation; MFC contributed to the assembly, protein clustering and gene ontology analysis; IL carried out the NMR assay; AFBZ contributed to draft the manuscript; PJLT performed the DNA library preparation and sequencing; PM coordinated the sequencing experiment; JR participated in design of experiments and helped to write the manuscript; GAGP coordinated, conceived and designed the experiments, analyzed the data and wrote the manuscript. All authors read and approved the final manuscript.

## Supplementary Material

Additional file 1 **Table S1. ***P. acidipropionici* proteins identified by UPLC-ESI-Q/TOF. List of proteins identified using UPLC-ESI-Q/TOF experiments. Click here for file

Additional file 2 **Figures S1 to S5.S1.** Comparative protein clustering. Four-set Venn diagram with protein families including *P. acidipropionici*, *P. acnes*, *P. freudenreichii* and *M. phosphovorus*. **S2.** Catabolic Repression in *P. acidipropionici*. Growth of *P. acidipropionici* in different carbon sources with or without addition of the hexose analogous 2-deoxy-glucose (2-DG). Error bars represent standard errors of the mean. **S3.** Alignment of CcO subunit I with homologs. Alignment of cytochrome C oxidase subunit I proteins of *Propionibacterium acidipropionici* ATCC4875 (Pacidi_2fr), *P. avidum* ATCC25577 (Pavidum; ZP_08938124.1), *P. acnes* KPA171202 (Pacnes; YP_055417.1) and *Microlunatus phosphovorus* NM-1 (Mphosp; YP_004574620.1). **S4.** CcO subunit I frameshift region. Alignment of 454 and Illumina reads with the frameshift region of cytochrome C oxidase subunit I. The combination of both technologies avoids the commom 454 homopolimer error, supporting the existence of the frameshift. First ten reads are from 454 sequencing. Last ten reads are from Illumina sequencing. **S5.** Gene clusters comprising B12 biosynthesis genes. The small cluster (red) harbors genes responsible for providing aminolaevulinic acid and converting it to uroporphyrinogen III, as well as the interconversion of uroporphyrinogen III into haem. The large cluster (green) harbors genes responsible for cobalt transport and adenosylcobalamin synthesis from uroporphyrinogen III. Click here for file

Additional file 3 **Table S2.** Transporter proteins comparison. Complete list of transporter families identified by annotation with TCDB classification. Click here for file

Additional file 4 **Table S3.** PTS protein-coding genes. List of annotated genes related to phosphotransferase system in *P.acidipropionici* genome. Click here for file
