# Supplementary material for: The genome sequence of Propionibacterium acidipropionici provides insights into its biotechnological and industrial potential
Source: BMC Genomics. 2012 Oct 19;13:562. doi: 10.1186/1471-2164-13-562 (PMC3534718; doi:10.1186/1471-2164-13-562)
Supplement: Additional file 1 — Table S1. P. acidipropionici proteins identified by UPLC-ESI-Q/TOF. List of proteins identified using UPLC-ESI-Q/TOF experiments. [file 1471-2164-13-562-S1.pdf]

Gene	Functional annotation	Mr (kDa)	Score	Number of peptides	Sequence coverage (%)
PACID_00010	Chromosomal replication initiator protein DnaA	55.0	62	2	9.2
PACID_00050	DNA polymerase III, beta chain	41.0	76	3	7.0
PACID_00100	DNA gyrase subunit B	76.3	109	4	4.7
PACID_00110	DNA gyrase subunit A	98.5	420	6	8.4
PACID_00120	putative membrane protein	23.5	83	2	10.1
PACID_00170	ribose ABC transport system protein	32.1	413	4	22.0
PACID_00180	probable ribokinase protein	30.5	301	5	6.5
PACID_00390	L-lactate dehydrogenase	36.6	284	4	11.7
PACID_00400	Pyruvate carboxylase	121.4	2096	46	28.8
PACID_00410	Methionine synthase II (Cobalamin-independent)	42.7	341	6	10.7
PACID_00950	Modification methylase NaeI	36.2	50	2	10.6
PACID_00970	Glycerol-3-phosphate dehydrogenase	36.3	73	1	6.3
PACID_01090	Putative phenylalanine aminotransferase	43.7	75	2	5.5
PACID_01100	Phospholipase, patatin family	33.0	45	1	4.1
PACID_01180	Predicted sugar phosphate isomerase	30.8	170	3	16.3
PACID_01250	Type II/IV secretion system protein	56.5	74	5	4.8
PACID_01400	Formate--tetrahydrofolate ligase	60.5	49	2	6.5
PACID_01450	ABC transporter, ATP-binding protein	32.7	254	4	10.4
PACID_01500	RtcB-like protein	43.3	296	5	16.6
PACID_01690	UDP-glucose 4-epimerase	35.9	302	5	26.1
PACID_01860	Lipoprotein A-like protein	28.8	158	5	12.5
PACID_02130	hypothetical protein	11.2	335	7	32.0
PACID_02150	Putative Acetate-CoA Ligase (ADP-forming)	75.1	1498	22	18.9
PACID_02230	Succinate-semialdehyde dehydrogenase	52.4	101	2	6.7
PACID_02270	Phosphofructokinase	39.7	252	10	14.6
PACID_02360	hypothetical protein	17.7	96	2	22.8
PACID_02400	starvation-inducible DNA-binding protein or fine tangled pili major subunit	19.5	3062	61	58.2
PACID_02460	GCN5-related N-acetyltransferase	10.8	185	7	27.4
PACID_02490	Glutathione transferase	39.1	133	2	8.7
PACID_02500	Glutathione S-transferase domain-containing protein	40.9	1096	21	28.5

PACID_02510	Glutathione transferase	37.8	119	1	5.1
PACID_02840	Lactonase drp35	34.2	332	4	14.5
PACID_02900	Sugar ABC transporter, solute-binding protein	37.7	90	1	8.1
PACID_02960	Dipeptidase	56.3	93	5	9.5
PACID_03050	Oxidoreductase	30.5	193	4	12.8
PACID_03120	Alpha-galactosidase	78.7	59	2	6.0
PACID_03360	AAA family ATPase	45.7	97	6	10.7
PACID_03610	Substrate-binding region of ABC-type glycine betaine transport system	35.3	617	14	30.2
PACID_03860	Pyridine nucleotide-disulphide oxidoreductase	48.4	426	11	28.2
PACID_03910	Beta-galactosidase	115.8	134	4	2.8
PACID_03970	putative general stress protein	17.0	235	7	12.4
PACID_04290	hypothetical protein	11.5	246	4	44.2
PACID_04450	alpha/beta hydrolase fold protein	26.6	174	5	6.2
PACID_04710	Bacterial transferase hexapeptide repeat protein	21.8	138	2	13.3
PACID_04720	DegT/DnrJ/EryC1/StrS aminotransferase family protein	39.3	895	17	29.2
PACID_04730	Oxidoreductase, NAD-binding domain-containing protein	35.8	476	13	44.5
PACID_04760	Phosphocarrier, HPr family	8.5	347	6	21.2
PACID_04850	Nucleotide sugar dehydrogenase	47.5	329	6	13.9
PACID_04870	Glycosyltransferase, group 1 family	42.1	137	4	10.1
PACID_04880	UDP-N-acetylglucosamine 2-epimerase	38.7	71	2	11.5
PACID_04960	Pyridine nucleotide-disulfide oxidoreductase	48.3	949	10	21.6
PACID_04990	Glycosyltransferase, group 1 family protein	43.1	132	3	9.2
PACID_05070	Thioredoxin reductase	35.2	73	2	11.6
PACID_05080	thioredoxin	11.6	1260	19	45.7
PACID_05120	Heavy metal-associated domain-containing protein	19.1	241	4	24.9
PACID_05140	Lipoprotein A-like protein	40.6	104	3	6.1
PACID_05160	Dihydroorotate dehydrogenase	36.0	208	5	14.3
PACID_05170	Pyruvate-flavodoxin oxidoreductase	132.3	875	13	9.2
PACID_05180	Pyridine nucleotide-disulfide oxidoreductase family protein associated with PFOR	67.3	320	8	9.7
PACID_05240	Iron-sulfur cluster-binding protein	57.3	446	9	17.6
PACID_05250	hypothetical protein	24.0	573	10	26.5
PACID_05380	Beta-galactosidase	64.4	49	2	8.1
PACID_05390	Extracellular solute-binding protein family 1	48.2	647	10	11.1

PACID_05650	hypothetical protein	13.5	103	3	8.1
PACID_05720	FHA domain-containing protein	17.9	150	4	7.3
PACID_05730	FHA domain-containing protein	25.4	78	2	10.8
PACID_05800	Zinc-binding dehydrogenase	32.0	3106	77	30.9
PACID_05880	Malto-oligosyltrehalose synthase	93.6	70	3	9.3
PACID_06030	DNA polymerase III, subunit gamma and tau	103.5	61	1	3.5
PACID_06200	aspartate kinase, monofunctional	44.8	89	2	8.7
PACID_06230	Polysaccharide deacetylase	56.2	94	3	7.6
PACID_06240	Possible tellerium resistance protein	37.3	108	3	10.4
PACID_06380	Raf-like protein	16.4	93	2	19.0
PACID_06420	Glucose-6-phosphate isomerase	60.9	1459	29	28.0
PACID_06440	Substrate-binding region of ABC-type glycine betaine transport system	35.6	217	5	8.6
PACID_06460	Oxidoreductase, aldo/keto reductase family protein	29.2	952	20	29.3
PACID_06470	Arginyl-tRNA synthetase	60.4	635	8	13.7
PACID_06490	ROK family protein	30.9	468	8	25.5
PACID_06620	hypothetical protein	10.5	72	2	17.3
PACID_06760	pyrroline-5-carboxylate reductase	27.0	137	2	10.5
PACID_06950	Propionyl-CoA:succinate coenzyme A transferase	55.3	1122	18	32.2
PACID_07000	Pyruvate, phosphate dikinase	97.4	1270	20	21.1
PACID_07020	FHA domain-containing protein	51.3	212	3	8.7
PACID_07060	Two component system response regulator	27.0	104	2	14.0
PACID_07090	Chaperone protein DnaK	66.6	2946	60	38.9
PACID_07100	Protein grpE	23.8	539	11	17.6
PACID_07280	Fructose-1,6-bisphosphate aldolase	32.5	313	8	27.9
PACID_07360	ATP-dependent chaperone protein ClpB	94.6	1003	25	27.2
PACID_07440	Heat shock protein 20 2 (20 kDa chaperone 2)	19.7	123	2	13.5
PACID_07620	Glycine betaine/L-proline transport ATP binding subunit	49.5	673	12	30.1
PACID_07640	ABC transporter, substrate-binding protein, QAT family	36.5	306	10	24.6
PACID_07650	ABC transporter, substrate-binding protein, QAT family	36.4	745	9	20.6
PACID_07750	Predicted aminotransferase	43.5	213	4	9.5
PACID_07790	LemA family protein	29.4	100	4	12.7
PACID_07960	Fructose-bisphosphate aldolase, class II	36.7	2029	60	69.5
PACID_07970	Methylmalonyl-CoA:Pyruvate transcarboxylase 5S subunit	54.9	4160	82	54.1

PACID_07980	Biotin dependent transcarboxylase 12S subunit	56.9	4431	75	55.3
PACID_07990	C-terminal of methylmalonyl-CoA carboxyltransferase	9.0	5512	92	69.1
PACID_08000	Biotin dependent transcarboxylase 1.3S subunit	11.7	11681	291	69.4
PACID_08010	Oxidoreductase, short chain dehydrogenase/reductase family protein	26.6	89	4	27.8
PACID_08020	Phosphoenolpyruvate-protein phosphotransferase	58.0	1291	17	18.0
PACID_08030	Phosphocarrier, HPr family	8.9	185	5	33.0
PACID_08040	2,3-bisphosphoglycerate-dependent phosphoglycerate mutase	28.2	1000	22	55.0
PACID_08050	Phosphate transport system regulatory protein PhoU	24.6	216	4	14.9
PACID_08140	PTS system, Lactose/Cellobiose specific IIB subunit	10.3	451	8	35.1
PACID_08150	Glycerol-3-phosphate dehydrogenase	37.8	1922	39	22.7
PACID_08200	PTS system glucitol/sorbitol-specific IIA component	12.8	161	7	44.9
PACID_08220	Oxidoreductase, putative	46.1	2043	76	41.0
PACID_08240	D-3-phosphoglycerate dehydrogenase	38.1	500	14	17.4
PACID_08260	Ketose-bisphosphate aldolase	30.8	1325	16	43.0
PACID_08270	hypothetical protein	19.8	1492	11	27.1
PACID_08280	Triose-phosphate isomerase	24.7	973	15	25.0
PACID_08320	hypothetical protein	62.8	79	3	6.0
PACID_08340	Serine hydroxymethyltransferase	52.7	63	1	6.1
PACID_08400	RNA methyltransferase, TrmH family, group 3	33.9	177	2	10.7
PACID_08410	Cysteinyl-tRNA synthetase	52.8	278	3	11.9
PACID_08430	hypothetical protein	49.3	226	3	12.4
PACID_08440	Cystathionine gamma-synthase (O-succinylhomoserine (Thiol)-lyase)	41.0	302	6	7.2
PACID_08450	Alkaline shock protein 23	20.4	323	5	14.2
PACID_08530	ABC transporter ATP-binding protein	39.6	1835	31	34.5
PACID_08830	Precorrin-3B C(17)-methyltransferase	90.2	63	2	6.9
PACID_08840	Sirohydrochlorin cobaltochelatase	47.6	390	5	9.1
PACID_08960	Nicotinate-nucleotide--dimethylbenzimidazole phosphoribosyltransferase	38.2	220	4	13.5
PACID_08970	Nicotinate-nucleotide--dimethylbenzimidazole phosphoribosyltransferase	21.0	59	2	10.1
PACID_09020	S-ribosylhomocysteine lyase	17.3	275	5	19.5
PACID_09060	60 kDa chaperonin 1	56.8	29384	506	87.8
PACID_09240	Phosphoserine aminotransferase	40.2	175	4	9.1
PACID_09250	N-acetylglucosamine-6-phosphate deacetylase	39.7	293	6	17.3
PACID_09270	Methyltransferase type 11	30.4	57	1	5.4

PACID_09380	putative myo-inositol 2-dehydrogenase	35.5	68	2	13.7
PACID_09400	UTP--glucose-1-phosphate uridylyltransferase	50.8	670	13	19.4
PACID_09460	MoeA N-terminal region (Domain I and II)	44.4	637	6	16.4
PACID_09650	3-oxoacyl-(Acyl-carrier-protein) synthase 2	40.8	2356	62	32.4
PACID_09700	intracellular protease, PfpI family protein	19.2	560	13	57.9
PACID_09730	Glyceraldehyde-3-phosphate dehydrogenase, type I	35.2	454	8	18.3
PACID_09740	Response regulator containing a CheY-like receiver domain and an HTH DNA-binding domain	23.1	176	4	37.1
PACID_09790	hypothetical protein	7.2	613	19	86.8
PACID_09930	Bifunctional protein glmU	51.7	275	6	9.0
PACID_09940	Ribose-phosphate pyrophosphokinase	35.3	281	7	20.8
PACID_09950	50S ribosomal protein L25	22.0	647	9	27.3
PACID_10000	enolase	45.5	15506	267	59.9
PACID_10170	Translation initiation factor IF-3	24.1	188	3	21.7
PACID_10180	rpmI multi-domain protein	7.4	115	3	30.9
PACID_10190	50S ribosomal protein L20	13.9	135	5	24.4
PACID_10230	Phenylalanyl-tRNA synthetase beta chain	89.2	164	2	5.2
PACID_10240	Biotin synthase	39.2	500	12	37.3
PACID_10270	Tyrosyl-tRNA synthetase	46.0	406	5	11.0
PACID_10350	Peptide methionine sulfoxide reductase MsrA	23.3	76	2	7.2
PACID_10380	hypothetical protein	35.8	296	5	18.6
PACID_10390	Transcription elongation factor greA	17.8	174	4	23.0
PACID_10410	hypothetical protein	6.8	138	3	28.1
PACID_10430	Undecaprenyl pyrophosphate synthase	30.9	48	7	8.4
PACID_10480	Exodeoxyribonuclease 7 large subunit	45.5	94	3	15.7
PACID_10540	GTP-binding protein YchF	39.5	923	15	11.3
PACID_10990	O-6-methylguanine DNA methyltransferase	21.2	207	2	12.4
PACID_11110	ACT domain-containing protein	18.0	61	2	19.2
PACID_11130	Oxidoreductase, aldo/keto reductase family protein	34.1	299	9	21.7
PACID_11140	Thiol peroxidase	17.2	606	7	64.7
PACID_11160	Alpha amylase, catalytic domain-containing protein	46.6	85	2	2.6
PACID_11200	Methylmalonyl-CoA mutase, small subunit	68.4	3365	58	32.9
PACID_11210	Methylmalonyl-CoA mutase	79.5	1319	25	20.7
PACID_11220	LAO/AO transport system ATPase	36.8	126	2	8.5

PACID_11370	Permease, cytosine/purine, uracil, thiamine, allantoin family	50.9	139	3	8.9
PACID_11390	Oxidoreductase, zinc-binding dehydrogenase family protein	37.0	307	4	13.0
PACID_11400	DAK1 domain-containing protein	58.1	143	6	7.4
PACID_11410	Phosphoribosyl transferase domain-containing protein	18.3	193	4	12.6
PACID_11430	Ribose 5-phosphate isomerase	17.2	125	4	16.7
PACID_11440	hypothetical protein	37.7	193	3	6.4
PACID_11450	HAD hydrolase, family IIA	31.6	47	1	3.5
PACID_11460	FGGY-family pentulose kinase	56.7	244	5	12.7
PACID_11470	3-dehydroquinate synthase	49.8	664	13	22.5
PACID_11480	Ribulokinase	62.8	375	6	9.0
PACID_11490	Transketolase	73.3	1186	20	19.8
PACID_11560	NAD(P) transhydrogenase subunit alpha	53.5	599	11	13.2
PACID_11570	NAD(P) transhydrogenase subunit beta	50.4	113	2	5.5
PACID_11590	VanW family protein	61.7	118	2	3.1
PACID_11600	Ferredoxin family protein	12.0	190	2	15.1
PACID_11640	2,3,4,5-tetrahydropyridine-2,6-dicarboxylate N-succinyltransferase	33.4	377	12	28.3
PACID_11650	Succinyl-diaminopimelate desuccinylase	39.4	75	2	10.5
PACID_11680	hypothetical protein	5.8	1611	29	65.5
PACID_11690	Glycogen synthase	43.9	90	2	7.2
PACID_11700	Glucose-1-phosphate adenylyltransferase	45.1	377	7	13.5
PACID_11730	O-methyltransferase	23.3	170	2	6.9
PACID_11750	MttA/ family protein	16.0	201	4	16.7
PACID_11760	ParA/MinD ATPase-like protein	40.5	88	2	8.1
PACID_11820	Peptidase, M24 family	56.1	134	2	4.8
PACID_11880	Zn-ribbon protein possibly nucleic acid-binding-like protein	27.6	166	2	14.6
PACID_11910	Methionine aminopeptidase	29.0	52	2	7.3
PACID_11930	Glutamine synthetase type I	49.9	132	4	9.2
PACID_11950	Glutamate-ammonia-ligase adenylyltransferase	107.5	72	2	3.9
PACID_11970	NGG1-interacting factor 3	28.9	170	2	9.6
PACID_12010	Glutamine synthetase	53.4	1488	22	37.2
PACID_12080	Integral membrane protein	28.5	108	2	10.6
PACID_12130	2-oxoglutarate dehydrogenase, E2 component, dihydrolipoamide succinyltransferase	46.2	1338	20	27.9
PACID_12170	PspA/IM30 family protein	28.5	639	7	25.2

PACID_12200	Iron binding protein from the HesB_IscA_SufA family protein	12.8	279	4	22.9
PACID_12240	IMP dehydrogenase family protein	52.4	403	6	12.7
PACID_12300	Response regulator receiver protein	14.7	57	2	16.7
PACID_12310	Anthranilate phosphoribosyltransferase	36.4	189	6	22.1
PACID_12340	putative regulatory protein	36.7	3454	73	34.3
PACID_12350	Peptidyl-prolyl cis-trans isomerase	18.4	297	5	20.0
PACID_12370	Putative transcriptional regulator	9.9	259	3	49.5
PACID_12420	Acyltransferase	28.2	121	3	14.8
PACID_12430	Phospholipid/glycerol acyltransferase	29.9	101	1	12.3
PACID_12470	Metallo-beta-lactamase family protein	23.3	190	3	16.7
PACID_12610	Hsp20/alpha crystallin family protein	17.2	63	2	14.6
PACID_12690	Glycine dehydrogenase (decarboxylating)	105.8	70	2	7.2
PACID_12700	Glycine cleavage system H protein	13.3	269	5	10.4
PACID_12710	Aminomethyltransferase	40.1	72	2	4.3
PACID_12750	ATPase family associated with various cellular activities (AAA)	34.4	1110	21	34.4
PACID_12800	UDP-N-acetylmuramoylalanyl-D-glutamate--2, 6-diaminopimelate ligase	55.3	61	2	11.9
PACID_12830	UDP-N-acetylmuramoylalanine--D-glutamate ligase	52.4	85	3	5.1
PACID_12850	UDP-N-acetylglucosamine--N-acetylmuramyl-(pentapeptide) pyrophosphoryl-undecaprenol N-acetylglucosamine transferase	40.1	192	6	10.7
PACID_12880	Cell division protein ftsZ	41.4	110	2	13.8
PACID_12920	DivIVA domain-containing protein	39.8	2473	49	61.8
PACID_12930	C4-type zinc finger protein, DksA/TraR family	16.0	121	2	13.2
PACID_12960	Pyruvate kinase	51.7	10715	182	53.4
PACID_12980	Response regulator receiver domain-containing protein	21.2	517	7	18.8
PACID_13000	Precorrin-6A synthase (Deacetylating)	28.0	201	3	12.3
PACID_13010	DNA polymerase I	97.2	534	8	7.4
PACID_13020	FAD linked oxidase protein	102.9	1998	34	23.8
PACID_13040	30S ribosomal protein S1	55.5	1340	30	35.8
PACID_13160	hypothetical protein	31.9	80	2	8.9
PACID_13170	CofD_related multi-domain protein	36.5	97	2	8.7
PACID_13190	Glyceraldehyde-3-phosphate dehydrogenase, type I	36.0	7154	138	86.0
PACID_13200	Phosphoglycerate kinase	41.8	2052	50	59.7
PACID_13210	Triosephosphate isomerase	27.6	860	16	45.6

PACID_13260	Nucleoside diphosphate kinase	16.2	49	2	6.9
PACID_13290	Radical SAM-linked protein	26.4	65	3	7.0
PACID_13300	Ribonuclease, Rne/Rng family	97.4	677	8	9.8
PACID_13310	50S ribosomal protein L21	9.4	183	4	43.7
PACID_13320	50S ribosomal protein L27	9.6	356	13	62.9
PACID_13350	Glutamate 5-kinase	41.1	108	3	22.6
PACID_13380	Nicotinate-nucleotide adenylyltransferase	23.9	76	1	11.6
PACID_13390	Iojap-like protein	14.7	84	2	11.3
PACID_13770	Leucine--tRNA ligase	93.9	156	4	2.7
PACID_13820	30S ribosomal protein S20	9.8	92	3	11.4
PACID_13920	Naphthoate synthase	36.2	110	3	4.0
PACID_14030	Extracellular solute-binding protein family 1	46.5	50	1	9.5
PACID_14060	Inositol monophosphatase family protein	29.4	69	2	3.7
PACID_14100	Chaperone protein DnaJ 1	41.6	829	9	12.3
PACID_14110	RNA methyltransferase, RsmE family	25.5	69	3	13.6
PACID_14120	Alcohol dehydrogenase GroES domain-containing protein	38.0	221	5	8.9
PACID_14140	DoxX protein	14.1	1557	31	63.5
PACID_14260	Proline iminopeptidase	37.3	93	2	6.9
PACID_14300	hypothetical protein	11.3	208	3	29.2
PACID_14310	Phosphate starvation-inducible protein PhoH, predicted ATPase	41.8	90	1	5.2
PACID_14380	2-isopropylmalate synthase	65.4	107	3	9.7
PACID_14890	ATPase family associated with various cellular activities (AAA)	40.2	93	2	6.3
PACID_14950	3-oxoacyl-[acyl-carrier-protein] synthase 1	44.0	1957	40	44.1
PACID_14960	Acyl carrier protein	8.9	4915	62	64.2
PACID_14970	3-oxoacyl-[acyl-carrier-protein] synthase 3	35.5	389	8	20.2
PACID_14980	[acyl-carrier-protein] S-malonyltransferase family protein	32.0	3372	56	62.7
PACID_15020	Pyruvate dehydrogenase E1 component	101.1	118	3	6.0
PACID_15050	GatB_Yqey, GatB/Yqey	16.3	148	2	15.7
PACID_15080	Carbamoyl-phosphate synthase, small subunit	42.5	97	1	4.6
PACID_15120	Orotidine 5'-phosphate decarboxylase	24.3	67	2	5.5
PACID_15140	hypothetical protein	18.4	59	2	8.5
PACID_15250	Protein RecA	37.8	792	12	24.9
PACID_15270	HDIG/KH domain-containing protein	55.3	68	4	7.6

PACID_15280	Aldose 1-epimerase	33.2	492	7	24.9
PACID_15720	hypothetical protein	31.6	47	2	2.9
PACID_15890	hypothetical protein	8.9	63	3	15.9
PACID_15930	hypothetical protein	7.1	128	4	42.4
PACID_15950	Diaminopimelate epimerase	26.9	182	3	10.8
PACID_16010	Ribonucleoside-diphosphate reductase, adenosylcobalamin-dependent	105.3	203	5	12.5
PACID_16030	hypothetical protein	34.9	147	3	11.9
PACID_16040	Universal stress family protein	30.6	975	19	30.6
PACID_16080	RNA polymerase sigma factor	51.0	127	2	8.7
PACID_16130	Med15 domain-containing protein	49.4	312	5	7.0
PACID_16140	Carbonate dehydratase	16.6	46	2	6.2
PACID_16150	DNA gyrase/topoisomerase IV, A subunit	90.7	84	3	9.4
PACID_16160	ABC transporter, ATP-binding protein	39.4	49	2	2.0
PACID_16180	Oligopeptide transporter, OPT family	68.4	59	1	5.0
PACID_16220	hypothetical protein	11.2	83	3	22.4
PACID_16330	Aconitate hydratase 1	96.7	933	22	14.7
PACID_16510	Oxidoreductase, zinc-binding dehydrogenase family protein	38.2	77	2	9.9
PACID_16520	Threonyl-tRNA synthetase	76.9	540	12	10.3
PACID_16620	FHA domain-containing protein	19.1	102	2	11.4
PACID_16640	hypothetical protein	16.7	79	1	19.0
PACID_16670	Pyrophosphate--fructose-6-phosphate 1-phosphotransferase	44.4	1926	38	41.9
PACID_16690	Sua5/YciO/YrdC/YwlC family protein	23.0	55	2	12.1
PACID_16740	hypothetical protein	125.0	572	11	10.0
PACID_16770	PAC2 family protein	40.5	122	2	10.3
PACID_16880	Carbon starvation protein	83.5	114	2	4.4
PACID_16890	Dihydrolipoyl dehydrogenase	50.3	608	13	19.4
PACID_16940	hypothetical protein	12.8	278	6	19.3
PACID_16950	Glycosyltransferase, group 2 family protein	28.3	133	3	18.4
PACID_17010	2,5-diketo-D-gluconate reductase	30.2	272	5	17.3
PACID_17450	predicted redox protein, regulator of disulfide bond formation	14.6	100	3	13.5
PACID_17470	GTP-binding protein engA	49.4	45	1	10.6
PACID_17490	Prephenate dehydrogenase	38.7	68	2	8.8
PACID_17560	Ribulose-phosphate 3-epimerase	23.9	57	1	8.6

PACID_17570	tRNA/rRNA cytosine-C5-methylase	48.2	55	1	5.3
PACID_17590	Primosomal protein	71.5	59	3	5.8
PACID_17680	Guanylate kinase	12.4	57	2	6.1
PACID_17700	Integration host factor	11.6	133	2	21.5
PACID_17720	Elongation factor P	21.0	484	9	21.7
PACID_17740	3-dehydroquinate synthase	38.5	60	3	10.5
PACID_17760	Chorismate synthase	42.7	73	5	12.0
PACID_17800	Alanyl-tRNA synthetase	96.9	748	13	18.8
PACID_17870	Aspartyl-tRNA synthetase	66.8	979	23	15.9
PACID_17880	Alpha/beta hydrolase fold protein	30.5	112	1	9.3
PACID_17890	hypothetical protein	39.7	66	2	11.0
PACID_17920	Histidyl-tRNA synthetase	50.1	210	2	9.1
PACID_17940	ATPase	46.2	915	12	27.5
PACID_17950	Candidate threonine synthase	51.8	94	3	5.9
PACID_17980	Adenine phosphoribosyltransferase	19.0	205	4	32.4
PACID_18000	Translocase subunit SecD	61.4	82	1	9.5
PACID_18010	Translocase subunit	15.6	132	4	22.9
PACID_18050	DNA-binding regulatory protein, YebC/PmpR family	28.7	489	7	32.2
PACID_18090	Histidinol-phosphate aminotransferase	43.7	273	6	18.4
PACID_18120	DNA polymerase III alpha subunit	132.4	71	2	5.1
PACID_18250	Indole-3-glycerol phosphate synthase	28.6	75	1	18.2
PACID_18340	Glycyl-tRNA synthetase	55.4	565	14	17.3
PACID_18450	Trehalose_PPase multi-domain protein	18.7	262	4	30.4
PACID_18550	ABC transporter ATP binding	58.1	149	4	11.2
PACID_18580	Cysteine synthase	32.2	5556	106	83.9
PACID_18610	Pyridoxal biosynthesis lyase pdxS	31.8	287	4	16.5
PACID_18980	Aspartyl/glutamyl-tRNA(Asn/Gln) amidotransferase subunit B	54.7	87	2	7.6
PACID_19000	Aspartyl/glutamyl-tRNA(Asn/Gln) amidotransferase subunit C	10.6	357	4	24.2
PACID_19130	Phosphorylase	94.7	647	13	11.1
PACID_19140	Alpha amylase, catalytic domain-containing protein	80.5	62	2	4.7
PACID_19160	1,4-alpha-glucan-branching enzyme	74.2	205	3	5.4
PACID_19180	Phosphotransferase system, EIIB	71.2	297	9	14.6
PACID_19200	Beta-phosphoglucomutase family hydrolase	25.2	221	4	11.0

PACID_19210	Thioredoxin	28.7	572	10	32.6
PACID_19220	Phosphoglucomutase, alpha-D-glucose phosphate-specific	58.1	573	15	18.3
PACID_19260	Methylmalonyl-CoA epimerase	17.1	1249	27	66.7
PACID_19270	Putative adhesion/surface protein	47.9	214	8	22.5
PACID_19320	ATP:cob(I)alamin adenosyltransferase	23.8	818	11	28.3
PACID_19350	ATP synthase subunit beta	53.1	651	12	22.5
PACID_19360	ATP synthase gamma chain	33.5	150	2	12.2
PACID_19370	ATP synthase subunit alpha	59.3	433	11	19.6
PACID_19380	ATP synthase subunit delta	28.8	46	2	7.4
PACID_19390	ATP synthase subunit b	20.8	201	4	19.3
PACID_19450	Acyl carrier protein	9.3	197	5	16.3
PACID_19640	SUA5 related protein	28.8	89	2	15.2
PACID_19670	50S ribosomal protein L31	10.5	294	4	38.9
PACID_19680	Transcription termination factor Rho	73.7	218	7	12.2
PACID_19700	Homoserine dehydrogenase	47.3	515	5	23.7
PACID_19760	Oxoglutarate dehydrogenase (Succinyl-transferring), E1 component	138.0	149	4	5.6
PACID_19790	Transcription factor WhiB	9.6	81	1	33.7
PACID_19840	DoxX protein	20.5	223	5	31.4
PACID_19970	Putative thermostable beta-glucosidase (Glycoside hydrolase family 3)	85.1	123	4	5.7
PACID_20010	putative hydrolase	50.8	264	3	6.9
PACID_20100	Cold shock-like protein CspA	7.6	152	4	41.2
PACID_20110	Protein phosphatase	31.2	108	2	10.3
PACID_20120	ATP-binding protein	8.1	344	6	58.7
PACID_20130	hypothetical protein	24.5	191	6	15.8
PACID_20140	DEAD/DEAH box helicase	67.3	150	4	9.0
PACID_20150	Tryptophan--tRNA ligase	40.1	78	3	8.5
PACID_20170	PHP domain-containing protein	31.3	162	2	7.7
PACID_20180	hypothetical protein	17.1	524	8	33.3
PACID_20190	hypothetical protein	18.1	91	2	11.7
PACID_20210	Protein translocase subunit secA	103.9	215	7	8.6
PACID_20230	Ribosomal subunit interface protein	22.8	1358	16	25.9
PACID_20270	Two component transcriptional regulator, winged helix family	26.8	70	6	8.3
PACID_20290	hypothetical protein	23.3	75	2	5.9

PACID_20300	Argininosuccinate lyase	51.7	118	2	6.9
PACID_20310	Ornithine carbamoyltransferase	32.9	116	3	12.3
PACID_20370	Efflux ABC transporter, permease protein	33.1	226	4	5.3
PACID_20380	Cell division ATP-binding protein FtsE	25.3	442	6	35.1
PACID_20390	Peptide chain release factor 2	40.8	230	3	11.4
PACID_20570	Pyrimidine-nucleoside phosphorylase	44.4	57	2	11.1
PACID_20590	Branched-chain-amino-acid transaminase	39.6	448	10	18.5
PACID_20610	Putative secreted protein	54.3	376	6	13.3
PACID_20640	Ketol-acid reductoisomerase	37.6	510	8	24.6
PACID_20680	Histidine triad domain-containing protein	14.1	158	2	11.5
PACID_20730	hypothetical protein	8.4	145	3	35.4
PACID_20780	Peptidyl-prolyl cis-trans isomerase, FKBP-type	36.3	118	2	8.2
PACID_20790	Pseudouridine synthase	27.8	131	3	16.6
PACID_20840	CTP synthase	61.9	395	13	10.6
PACID_20900	BioY multi-domain protein	20.6	111	5	8.3
PACID_20910	HAD hydrolase, family IIA	34.7	73	3	9.9
PACID_20980	ATP phosphoribosyltransferase	31.0	333	5	19.2
PACID_20990	Phosphoribosyl-ATP pyrophosphatase	9.9	74	2	20.5
PACID_21030	Citrate (Si)-synthase	48.3	103	2	7.9
PACID_21110	Ribulose-5-phosphate 4-epimerase-like epimerase or aldolase	25.8	52	2	10.1
PACID_21230	Carbohydrate kinase, FGGY-like protein	56.7	82	4	14.8
PACID_21300	50S ribosomal protein L19	13.3	354	14	41.0
PACID_21310	Succinate dehydrogenase iron-sulfur protein	28.1	414	8	29.2
PACID_21320	Succinate dehydrogenase flavoprotein subunit	76.1	3992	72	30.3
PACID_21330	Succinate dehydrogenase cytochrome B-558 subunit	25.1	101	2	9.7
PACID_21360	KH domain-containing protein	9.4	201	5	51.2
PACID_21370	Ribosomal protein S16	14.6	659	15	48.1
PACID_21390	Signal recognition particle protein	56.8	174	4	9.5
PACID_21440	Glycosyl transferase, group 2 family	41.3	66	2	7.8
PACID_21450	Signal recognition particle-docking protein FtsY	40.9	613	13	29.0
PACID_21540	Ribonuclease 3	28.0	167	4	15.8
PACID_21550	50S ribosomal protein L32 1	7.3	487	9	49.2
PACID_21590	Pyridoxamine 5'-phosphate oxidase family protein	15.4	595	8	28.8

PACID_21620	50S ribosomal protein L28 1	7.2	74	3	36.1
PACID_21630	Zn-dependent hydrolase, RNA-metabolizing, CPSF	61.6	660	13	18.3
PACID_21650	Thiamine pyrophosphate enzyme	62.1	1791	34	32.8
PACID_21670	Dihydrodipicolinate reductase	26.0	311	6	24.8
PACID_21680	Polyribonucleotide nucleotidyltransferase	79.1	2208	37	27.1
PACID_21690	30S ribosomal protein S15	9.9	214	7	48.3
PACID_21830	Translation initiation factor IF-2	105.2	761	19	19.8
PACID_21900	Prolyl-tRNA synthetase	64.3	1003	16	19.5
PACID_22010	NADP-specific glutamate dehydrogenase	48.3	51	1	9.0
PACID_22040	Ribosome-recycling factor	20.8	712	13	36.8
PACID_22050	Uridylate kinase	25.7	190	5	14.8
PACID_22060	Elongation factor Ts	28.4	1357	29	48.1
PACID_22070	30S ribosomal protein S2	33.5	1239	24	49.7
PACID_22230	Band 7 stomatin-like protein	47.8	502	7	11.2
PACID_22250	ABC transporter, ATP-binding protein	28.0	406	7	14.9
PACID_22280	Aspartate ammonia-lyase	52.2	74	14	9.7
PACID_22300	Enoyl-[acyl-carrier-protein] reductase (NADH)	27.4	2234	40	50.2
PACID_22310	3-oxoacyl-[acyl-carrier-protein] reductase	24.0	2104	26	58.0
PACID_22480	FeS assembly ATPase SufC	27.7	307	8	30.2
PACID_22490	Rieske [2Fe-2S] domain-containing protein	12.4	97	2	25.0
PACID_22500	FeS assembly protein SufD	45.7	53	2	6.2
PACID_22540	Bacterial extracellular solute-binding protein	43.5	1618	25	38.4
PACID_22630	ABC transporter ATP-binding protein	26.2	294	6	15.0
PACID_22670	Glucose-6-phosphate 1-dehydrogenase	58.0	90	2	6.8
PACID_22680	Putative OxPP cycle protein OpcA	36.4	176	4	8.8
PACID_22700	DsbA-like protein	28.6	59	2	9.1
PACID_22710	Valyl-tRNA synthetase	97.8	378	6	7.4
PACID_22740	WD40-like protein	120.5	79	4	6.8
PACID_22770	ATP-dependent Clp protease proteolytic subunit 1	24.7	457	10	25.3
PACID_22780	ATP-dependent Clp protease proteolytic subunit 2	22.7	213	4	18.4
PACID_22800	Trigger factor	52.1	2568	32	41.9
PACID_23070	Ribose 5-phosphate isomerase	17.3	165	4	19.2
PACID_23120	Aminopeptidase N	95.2	501	7	7.8

PACID_23140	6-phosphogluconate dehydrogenase, decarboxylating	51.4	1265	20	22.0
PACID_23220	FAD linked oxidase, C-terminal domain-containing protein	101.6	74	3	7.1
PACID_23230	ATP-binding cassette protein, ChvD family	62.2	1011	19	25.1
PACID_23270	Oligoribonuclease	22.7	94	1	17.2
PACID_23580	Nitroreductase	18.3	49	2	13.3
PACID_23670	Glyoxalase family protein	15.2	54	4	19.6
PACID_23770	hypothetical protein	21.5	115	2	16.1
PACID_23850	tRNA nucleotidyltransferase	26.4	189	4	4.9
PACID_23920	Nicotinate phosphoribosyltransferase	47.7	192	4	10.7
PACID_23930	DNA-binding protein HU	9.6	975	29	69.2
PACID_23940	4-alpha-glucanotransferase	78.8	78	7	5.4
PACID_23980	3-dehydroquinate dehydratase, type II	18.4	151	2	8.8
PACID_24090	Mannose-1-phosphate guanylyltransferase	38.9	463	9	21.5
PACID_24130	Phosphoribosylaminoimidazole carboxylase, catalytic subunit	20.6	68	2	9.2
PACID_24140	Phosphoribosylaminoimidazole carboxylase, ATPase subunit	46.0	63	3	7.7
PACID_24160	Peptidase family S51	24.9	215	7	11.3
PACID_24180	Carboxyl transferase domain-containing protein	57.2	51	2	12.8
PACID_24250	Hydroxymethylpyrimidine/phosphomethylpyrimidine kinase	27.8	902	18	39.0
PACID_24300	Purine nucleoside phosphorylase	28.3	230	3	18.8
PACID_24310	Phosphoglucomutase/phosphomannomutase, alpha/beta/alpha domain II	58.9	80	4	10.8
PACID_24440	Methionyl-tRNA synthetase	66.8	409	9	11.9
PACID_24540	Isocitrate dehydrogenase, NADP-dependent	80.3	536	9	8.5
PACID_24550	Formyltetrahydrofolate deformylase	32.5	52	2	6.2
PACID_24560	Malate dehydrogenase	34.2	4938	95	67.3
PACID_24580	Aldehyde dehydrogenase family protein	50.0	651	10	20.5
PACID_24600	Bifunctional protein FolD	30.2	591	8	27.8
PACID_24690	hypothetical protein	63.7	110	4	11.1
PACID_24700	Succinyl-CoA ligase [ADP-forming] subunit alpha	30.4	498	7	24.2
PACID_24710	Succinyl-CoA ligase [ADP-forming] subunit beta	41.1	1793	25	36.8
PACID_24760	ABC-type metal ion transport system, periplasmic component/surface antigen	33.8	487	10	26.8
PACID_24770	Methionine import ATP-binding protein MetN	37.1	176	6	13.8
PACID_24790	Cardiolipin synthetase	47.5	79	4	4.8
PACID_24850	PspC domain-containing protein	11.1	55	2	25.2

PACID_24860	GMP synthase (glutamine-hydrolyzing)	56.2	798	16	18.6
PACID_24870	Serine acetyltransferase	22.6	79	2	20.3
PACID_24900	IMP dehydrogenase family protein	39.5	102	2	10.6
PACID_24910	Inosine-5'-monophosphate dehydrogenase	55.3	700	11	12.0
PACID_24930	60 kDa chaperonin 2	56.0	31216	522	75.5
PACID_24940	10 kDa chaperonin	10.7	3558	96	88.8
PACID_24980	MEMO_like multi-domain protein	29.4	70	3	7.6
PACID_25020	Probable tRNA threonylcarbamoyladenosine biosynthesis protein Gcp	36.1	682	10	15.9
PACID_25080	YjeF C-terminal domain family protein	47.9	86	2	8.4
PACID_25100	Glucosamine--fructose-6-phosphate aminotransferase	66.9	686	14	17.7
PACID_25140	Phosphoglucosamine mutase	47.8	1078	21	35.1
PACID_25150	Oxidoreductase, short chain dehydrogenase/reductase family protein	24.4	513	14	39.1
PACID_25160	30S ribosomal protein S9	18.7	475	9	42.3
PACID_25170	50S ribosomal protein L13	16.2	465	11	46.9
PACID_25280	Superoxide dismutase	23.1	4002	87	70.9
PACID_25290	Glutamine amidotransferase class-I	24.5	167	4	10.9
PACID_25320	50S ribosomal protein L17	19.4	1008	26	51.4
PACID_25330	DNA-directed RNA polymerase subunit alpha	37.4	1160	23	34.6
PACID_25350	30S ribosomal protein S4	23.2	949	22	47.3
PACID_25360	30S ribosomal protein S11	14.2	449	9	34.1
PACID_25370	30S ribosomal protein S13	13.9	508	9	35.5
PACID_25390	Translation initiation factor IF-1	8.4	715	15	54.8
PACID_25430	Adenylate kinase	20.3	481	8	22.8
PACID_25500	50S ribosomal protein L15	15.4	671	16	44.5
PACID_25510	50S ribosomal protein L30	6.7	109	5	25.0
PACID_25520	30S ribosomal protein S5	22.2	1061	19	47.7
PACID_25530	50S ribosomal protein L18	13.6	261	8	42.5
PACID_25540	50S ribosomal protein L6	19.7	804	17	57.8
PACID_25550	30S ribosomal protein S8	14.6	1525	31	55.6
PACID_25570	50S ribosomal protein L5	23.6	538	20	45.2
PACID_25580	50S ribosomal protein L24	13.3	529	19	65.6
PACID_25590	50S ribosomal protein L14	13.5	359	10	53.3
PACID_25670	30S ribosomal protein S17	10.5	309	17	58.2

PACID_25680	50S ribosomal protein L29	8.9	534	10	62.0
PACID_25690	50S ribosomal protein L16	15.8	1545	15	33.1
PACID_25700	30S ribosomal protein S3	29.9	1812	30	42.3
PACID_25710	50S ribosomal protein L22	16.9	597	12	39.6
PACID_25720	30S ribosomal protein S19	10.5	414	14	55.9
PACID_25730	50S ribosomal protein L2	30.0	175	12	29.9
PACID_25740	50S ribosomal protein L23	11.3	714	18	58.8
PACID_25750	50S ribosomal protein L4	31.6	5610	120	57.9
PACID_25760	50S ribosomal protein L3	23.7	2053	48	51.6
PACID_25770	30S ribosomal protein S10	11.7	403	10	46.6
PACID_25780	Glutamyl-tRNA synthetase	52.6	390	6	3.4
PACID_25830	Elongation factor Tu	44.1	21088	524	68.3
PACID_25850	Elongation factor G	77.0	3141	48	37.1
PACID_25860	30S ribosomal protein S7	17.5	1085	18	62.2
PACID_25870	30S ribosomal protein S12	15.5	143	6	23.7
PACID_25910	DNA-directed RNA polymerase subunit beta'	144.2	669	20	17.3
PACID_25920	DNA-directed RNA polymerase subunit beta	129.2	832	21	17.1
PACID_25930	50S ribosomal protein L7/L12	13.5	6252	166	51.6
PACID_25940	50S ribosomal protein L10	19.7	1349	20	60.9
PACID_25950	50S ribosomal protein L1	24.7	1846	32	39.2
PACID_25960	50S ribosomal protein L11	15.2	692	12	62.2
PACID_25970	Transcription antitermination protein nusG	34.5	347	7	21.8
PACID_26000	Aminotransferase, class I/II	46.2	267	5	15.4
PACID_26040	50S ribosomal protein L33	6.8	139	3	28.6
PACID_26150	2-oxoglutarate oxidoreductase, alpha subunit	65.9	201	3	9.1
PACID_26160	2-oxoglutarate ferredoxin oxidoreductase subunit beta	41.0	56	1	4.2
PACID_26170	Peptidase family M13	74.8	348	10	10.3
PACID_26270	Polyprenyl synthetase	34.7	71	2	6.8
PACID_26290	Proton-translocating NADH-quinone oxidoreductase, chain M	53.4	98	2	3.2
PACID_26320	NADH-ubiquinone/plastoquinone oxidoreductase chain 6	28.8	362	4	16.0
PACID_26330	NADH-quinone oxidoreductase subunit I	21.0	53	1	10.9
PACID_26340	NADH-quinone oxidoreductase subunit H	49.6	72	2	2.4
PACID_26350	NADH-quinone oxidoreductase	89.1	292	14	12.4

PACID_26360	NADH oxidoreductase (Quinone), F subunit	48.8	61	1	4.7
PACID_26370	NADH-quinone oxidoreductase, E subunit	28.1	78	1	13.7
PACID_26380	NADH-quinone oxidoreductase subunit D	49.2	150	4	6.6
PACID_26420	Geranylgeranyl reductase family protein	46.9	150	9	12.0
PACID_26480	Heme ABC exporter, ATP-binding protein CcmA	23.0	157	4	21.0
PACID_26490	Ubiquinone/menaquinone biosynthesis methyltransferase UbiE	25.3	45	1	6.0
PACID_26550	DhaK PTS-dependent dihydroxyacetone kinase, dihydroxyacetone-binding subunit	34.8	970	15	18.6
PACID_26560	Dihydroxyacetone kinase, L subunit	23.1	846	11	37.2
PACID_26570	Phosphoenolpyruvate-protein phosphoryltransferase PptE	15.6	138	3	21.1
PACID_26600	GHMP kinase, N-terminal domain-containing protein	42.7	49	3	2.7
PACID_26640	Predicted sugar phosphatase/hydrolase	28.6	798	12	27.8
PACID_26690	Mevalonate kinase	37.1	74	2	7.9
PACID_26710	Phosphomevalonate kinase	39.2	80	3	6.1
PACID_26980	NAD dependent epimerase/dehydratase family protein	22.3	508	12	40.3
PACID_27020	Putative branched-chain amino acid ABC transporter, substrate-binding protein	44.5	275	3	13.2
PACID_27120	Phosphoribosylformylglycinamidine cyclo-ligase	37.7	512	10	6.1
PACID_27130	Amidophosphoribosyltransferase	54.5	84	2	8.1
PACID_27150	Peptidase dimerization domain-containing protein	47.3	1234	24	20.0
PACID_27190	Stomatin/prohibitin-like protein	28.5	88	2	14.7
PACID_27200	Phosphoribosylformylglycinamidine synthase 2	79.8	292	4	9.3
PACID_27250	Membrane alanyl aminopeptidase	92.9	79	2	4.2
PACID_27280	Phosphoribosylaminoimidazole-succinocarboxamide synthase	50.8	116	5	11.0
PACID_27290	Peroxiredoxin	20.7	793	10	44.9
PACID_27320	Adenylosuccinate lyase	52.3	604	8	14.3
PACID_27330	Phosphoribosylamine--glycine ligase	45.3	613	8	23.3
PACID_27370	Adenylosuccinate synthetase	47.1	75	2	6.1
PACID_27380	Acetyltransferase, GNAT family	31.6	100	4	13.9
PACID_27390	hypothetical protein	25.2	52	1	8.6
PACID_27450	GTP-binding protein TypA	68.8	372	6	13.1
PACID_27500	hypothetical protein	5.7	93	1	30.6
PACID_27580	Polyphosphate kinase	79.6	629	11	14.9
PACID_27840	Mycothione reductase	51.8	303	4	6.6
PACID_27860	Dihydroxy-acid dehydratase	68.0	276	9	3.1

PACID_27900	Glycerol-3-phosphate dehydrogenase (NAD(P)+)	63.3	338	9	12.3
PACID_27910	Oxidoreductase, short chain dehydrogenase/reductase family protein	29.0	200	6	11.5
PACID_28040	Semialdehyde dehydrogenase	37.7	235	5	15.4
PACID_28080	Glycosyltransferase	23.2	570	10	29.7
PACID_28110	DNA integrity scanning protein DisA	39.4	48	2	5.0
PACID_28130	Ferrochelatase	76.2	167	4	7.6
PACID_28290	TIGR01777 family protein	32.8	171	4	19.7
PACID_28350	hypothetical protein	28.6	171	3	16.9
PACID_28360	hypothetical protein	32.3	135	4	9.2
PACID_28400	Negative regulator of genetic competence ClpC/MecB	92.4	703	16	15.8
PACID_28410	Lsr2-like protein	11.6	215	6	31.1
PACID_28490	ATP-dependent zinc metalloprotease FtsH	79.8	89	1	7.9
PACID_28530	Inorganic pyrophosphatase	20.4	102	5	21.2
PACID_28600	Nitrogen regulatory protein P-II	12.1	438	5	27.7
PACID_28610	von Willebrand factor type A	23.6	49	2	4.6
PACID_28700	Bacterial extracellular solute-binding protein, family 5	66.5	249	5	8.1
PACID_28710	Branched-chain amino acid aminotransferase	30.9	163	5	15.5
PACID_28730	Class I glutamine amidotransferase	83.2	69	2	2.7
PACID_28750	FAD dependent oxidoreductase	47.5	1238	18	20.5
PACID_28880	DNA topoisomerase	104.4	57	2	4.1
PACID_29090	hypothetical protein	16.8	319	4	24.4
PACID_29140	Endoribonuclease L-PSP	14.8	2977	65	31.8
PACID_29210	hypothetical protein	69.9	54	1	9.0
PACID_29300	Bacterial extracellular solute-binding protein, family 5	60.5	115	3	9.6
PACID_29380	Peptidyl-prolyl cis-trans isomerase	12.9	431	6	28.1
PACID_29500	hypothetical protein	7.8	302	6	34.7
PACID_29510	hypothetical protein	8.9	162	4	21.0
PACID_29690	hypothetical protein	10.0	144	5	35.1
PACID_29720	catalase/hydroperoxidase HPI(I)	81.1	60	2	2.0
PACID_29880	CRISPR-associated protein, Cse4 family	42.3	442	8	9.6
PACID_29900	Cse3 family CRISPR-associated protein	29.9	75	1	8.1
PACID_30370	Lipoprotein A-like double-psi beta-barrel	23.5	214	8	30.8
PACID_30450	hypothetical protein	11.2	84	3	23.7

PACID_30490	Putative ABC transporter ATP-binding subunit	40.9	191	2	10.1
PACID_30640	putative tRNA adenosine deaminase-associated protein	27.5	253	4	8.4
PACID_30650	Uracil phosphoribosyltransferase	23.3	146	3	21.6
PACID_30730	Oxidoreductase	35.7	144	3	9.8
PACID_30820	Galactokinase	44.8	52	2	4.5
PACID_30840	Sodium:solute symporter	60.4	305	8	6.2
PACID_30860	Membrane protein without function	17.9	89	6	12.6
PACID_30890	NAD(P)H quinone oxidoreductase, PIG3 family	34.8	127	4	18.9
PACID_31020	Seryl-tRNA synthetase	46.5	847	15	26.1
PACID_31550	Membrane protein	26.1	61	1	6.8
PACID_31570	Transcriptional regulator	33.0	187	2	5.8
PACID_31580	Carbohydrate kinase, FGGY	53.2	161	2	5.1
PACID_31590	Carbohydrate kinase, FGGY family protein	54.7	560	11	13.2
PACID_31600	4-phosphoerythronate dehydrogenase	38.8	776	14	18.1
PACID_31610	Putative L-ribulose-5-phosphate 4-epimerase	23.5	590	11	34.9
PACID_31630	Fructose-bisphosphate aldolase	29.4	603	10	33.8
PACID_31640	Alcohol dehydrogenase superfamily, zinc-containing	36.0	296	6	13.9
PACID_31770	Extracellular solute-binding protein family 1	46.0	98	1	9.4
PACID_31840	Glycoside hydrolase family 31	91.2	217	4	5.5
PACID_31950	hypothetical protein	12.6	136	3	14.4
PACID_31970	Transcriptional regulator/sugar kinase	40.4	47	2	9.1
PACID_32050	Putative ABC transporter permease/ATP-binding protein	68.1	273	3	5.0
PACID_32170	S51 family peptidase	47.7	172	6	6.2
PACID_32210	Isopentenyl-diphosphate Delta-isomerase	25.7	171	3	9.1
PACID_32240	YCII-related protein	10.3	69	2	16.7
PACID_32260	Oxidoreductase, FAD/FMN-binding protein	39.2	99	2	6.6
PACID_32320	50S ribosomal protein L9	16.0	716	17	51.4
PACID_32330	30S ribosomal protein S18	8.8	116	3	29.1
PACID_32340	Single-stranded DNA-binding protein	21.9	203	4	12.9
PACID_32350	30S ribosomal protein S6	11.3	424	14	26.0
PACID_32420	Apurinic endonuclease (APN1)	27.9	287	7	17.2
PACID_32440	Acyltransferase	34.4	156	6	12.5
PACID_32470	FemAB family protein	43.8	124	2	11.3

PACID_32500	Ferredoxin reductase	44.3	167	3	15.3
PACID_32690	Glycerol-3-phosphate dehydrogenase, anaerobic, C subunit	47.0	647	11	28.3
PACID_32700	Glycerol-3-phosphate dehydrogenase, anaerobic, B subunit	46.6	807	20	21.9
PACID_32710	Glycerol-3-phosphate dehydrogenase, anaerobic, A subunit	59.6	190	9	10.7
PACID_32730	4-phosphoerythronate dehydrogenase	34.0	318	8	15.0
PACID_32770	YceI-like protein	19.3	52	1	6.1
PACID_32840	TetR family transcriptional regulator	22.1	157	4	18.0
PACID_32960	LMBE-related protein	32.3	83	2	5.1
PACID_33110	Serine/threonine protein kinase	61.0	52	1	6.4
PACID_33280	Adenosine deaminase	38.1	45	1	8.5
PACID_33440	Fumarate hydratase, class II	50.8	2638	41	26.9
PACID_33480	Methyltransferase type 12	31.2	48	1	9.4
PACID_33490	Aminopeptidase C	49.6	745	10	17.3
PACID_33620	tRNA adenylyltransferase	57.4	46	2	2.7
PACID_33650	Putative zinc-binding oxidoreductase	37.1	170	9	10.8
PACID_33690	RNA polymerase, sigma subunit, ECF family	21.3	138	3	22.7
PACID_33710	Glycerol kinase	55.6	2699	52	47.5
PACID_33730	Glycerol uptake facilitator protein	26.0	107	2	8.0
PACID_33870	NAD-dependent epimerase/dehydratase	20.8	674	14	46.5
PACID_33910	OsmC-like protein	15.7	69	4	8.3
PACID_33940	Putative signal transduction histidine kinase	82.0	99	6	9.5
PACID_34180	hypothetical protein	25.2	62	2	4.7
PACID_34190	Aminotransferase, class I/II	49.3	55	1	8.3
PACID_34210	D-alanine--D-alanine ligase B	33.2	90	2	13.0
PACID_34260	Putative partitioning protein ParA	30.7	83	1	13.4
PACID_34300	R3H domain-containing protein	22.6	86	3	11.5
