# Supplementary material for: The genome sequence of Propionibacterium acidipropionici provides insights into its biotechnological and industrial potential
Source: BMC Genomics. 2012 Oct 19;13:562. doi: 10.1186/1471-2164-13-562 (PMC3534718; doi:10.1186/1471-2164-13-562)

Supplementary figures

Figure S1. Comparative protein clustering. Four-set Venn diagram with protein families including *P. acidipropionici*, *P. acnes*, *P. freudenreichii* and *M. phosphovorus*.

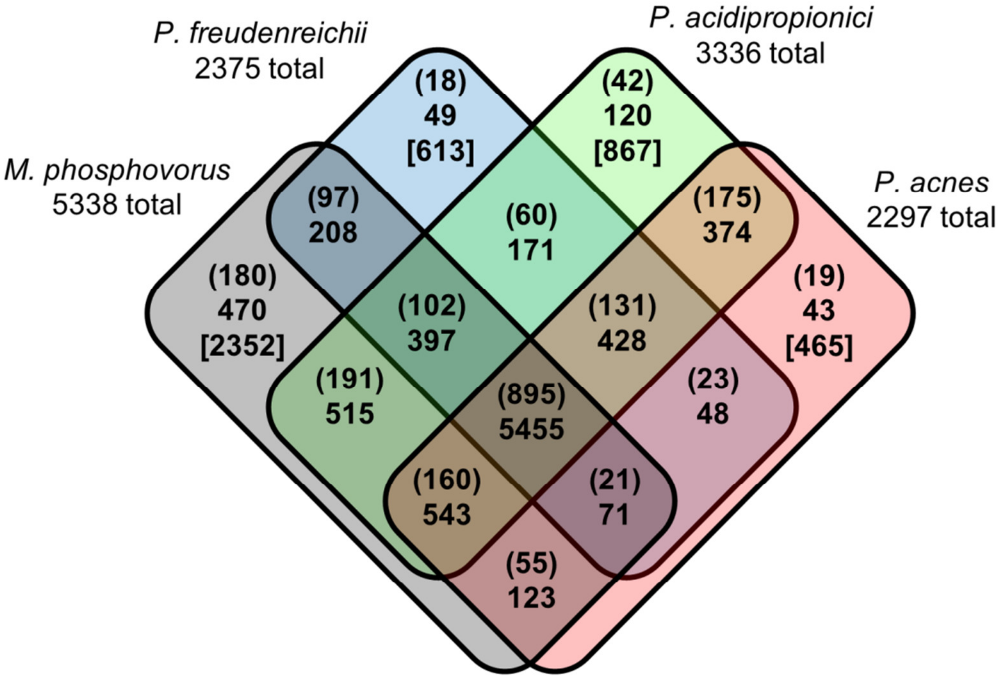

Figure S2. Catabolic Repression in *P. acidipropionici*. Growth of *P. acidipropionici* in different carbon sources with or without addition of the hexose analogous 2-deoxy-glucose (2-DG). Error bars represent standard errors of the mean.

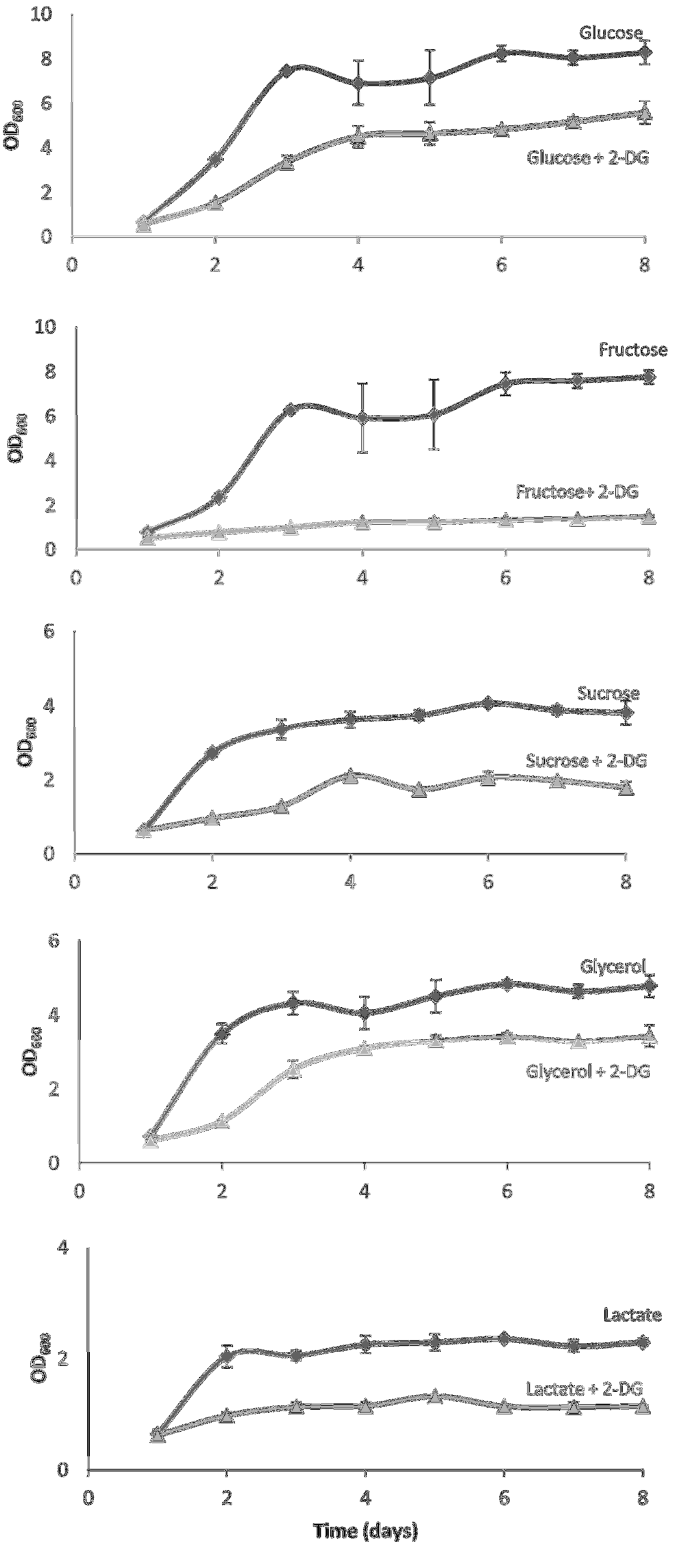

Glucose
Glucose + 2-DG
Fructose
Fructose+ 2-DG
Sucrose
Sucrose + 2-DG
Glycerol
Glycerol + 2-DG
Lactate
Lactate + 2-DG
OD_{600}
Time (days)

Figure S3. Alignment of CcO subunit I with homologs. Alignment of cytochrome C oxidase subunit I proteins of *Propionibacterium acidipropionici* ATCC4875 (Pacidi_2fr), *P. avidum* ATCC25577 (Pavidum; ZP_08938124.1), *P. acnes* KPA171202 (Pacnes; YP_055417.1) and *Microlunatus phosphovorus* NM-1 (Mphosp; YP_004574620.1).

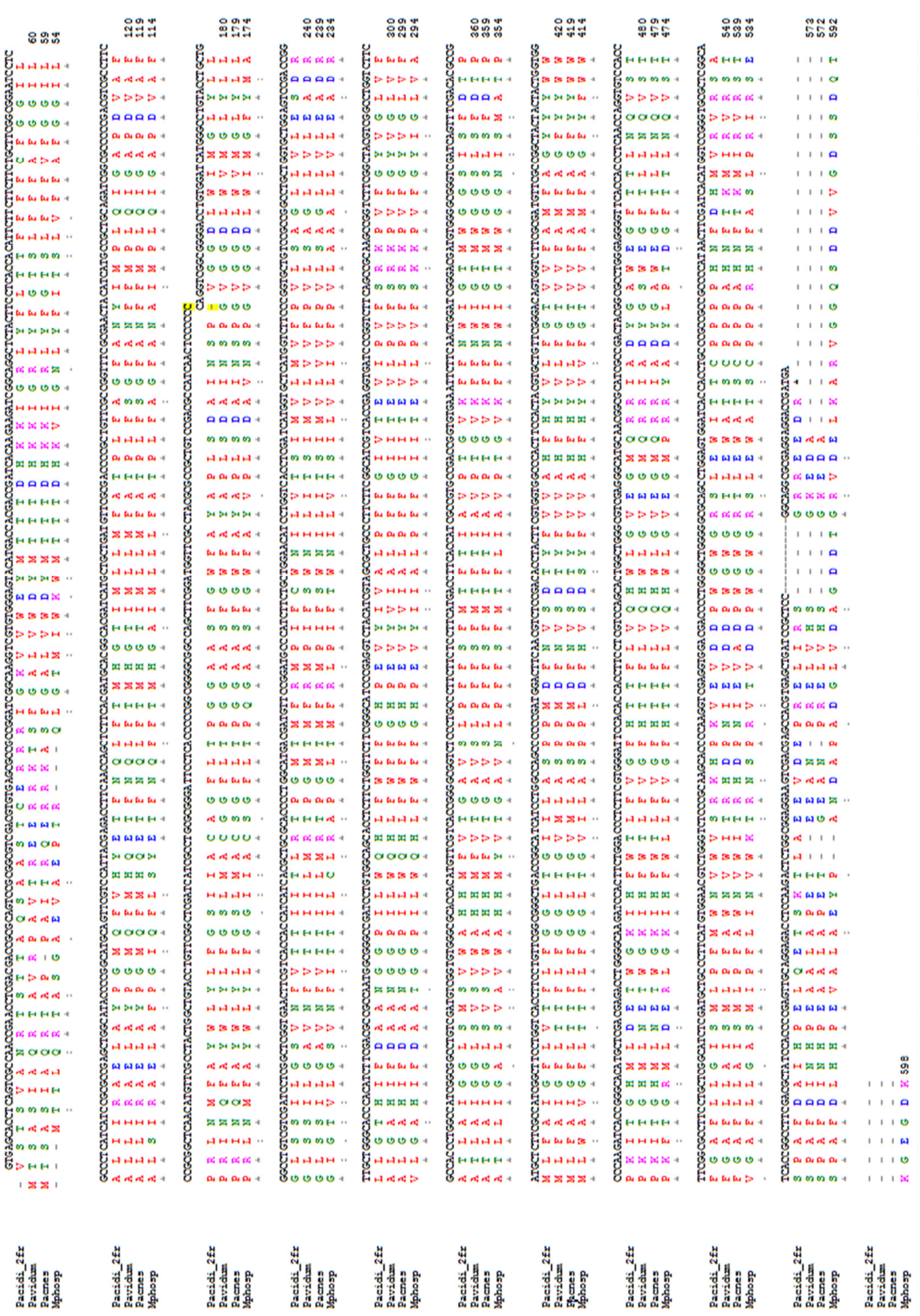

Figure S4. CcO subunit I frameshift region. Alignment of 454 and Illumina reads with the frameshift region of cytochrome C oxidase subunit I. The combination of both technologies avoids the commom 454 homopolimer error, supporting the existence of the frameshift. First ten reads are from 454 sequencing. Last ten reads are from Illumina sequencing.

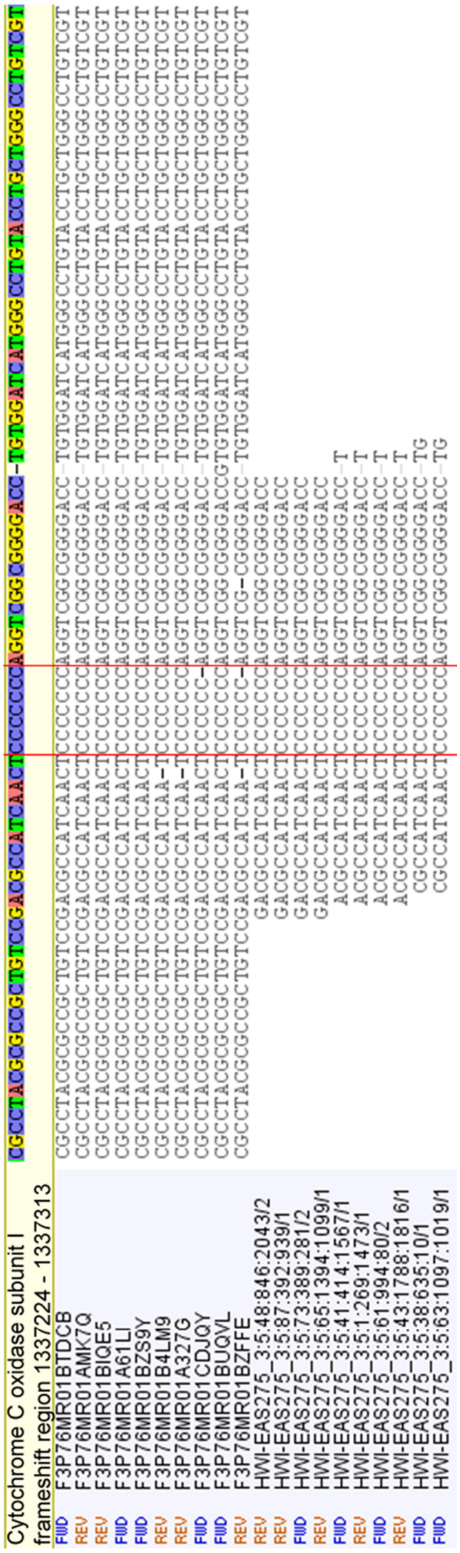

Figure S5. Gene clusters comprising B12 biosynthesis genes. The small cluster (red) harbors genes responsible for providing aminolaevulinic acid and converting it to uroporphyrinogen III, as well as the interconversion of uroporphyrinogen III into haem. The large cluster (green) harbors genes responsible for cobalt transport and adenosylcobalamin synthesis from uroporphyrinogen III.

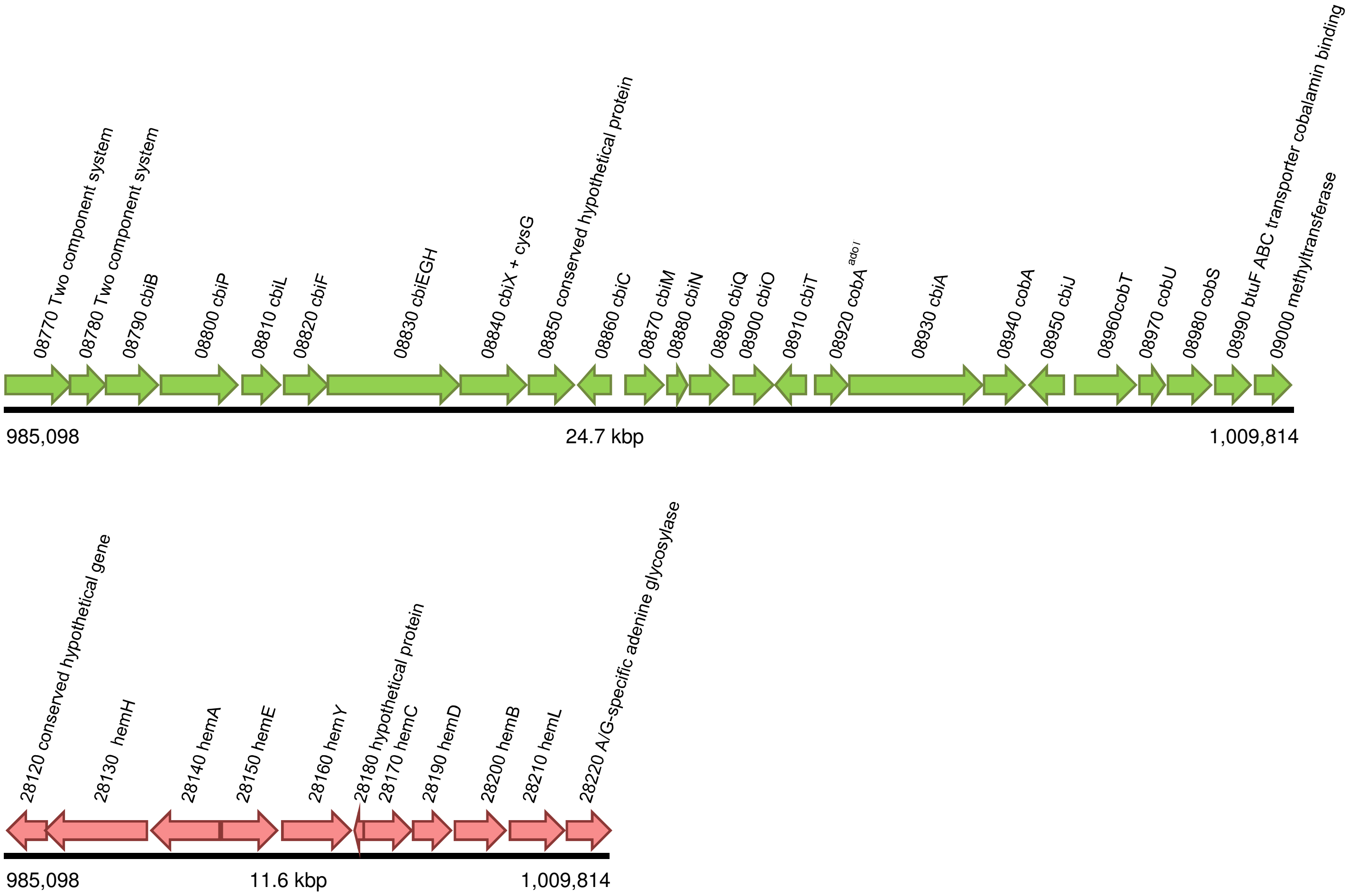

Supplement: Additional file 2 — Figures S1 to S5.S1. Comparative protein clustering. Four-set Venn diagram with protein families including P. acidipropionici, P. acnes, P. freudenreichii and M. phosphovorus. S2. Catabolic Repression in P. acidipropionici. Growth of P. acidipropionici in different carbon sources with or without addition of the hexose analogous 2-deoxy-glucose (2-DG). Error bars represent standard errors of the mean. S3. Alignment of CcO subunit I with homologs. Alignment of cytochrome C oxidase subunit I proteins of Propionibacterium acidipropionici ATCC4875 (Pacidi_2fr), P. avidum ATCC25577 (Pavidum; ZP_08938124.1), P. acnes KPA171202 (Pacnes; YP_055417.1) and Microlunatus phosphovorus NM-1 (Mphosp; YP_004574620.1). S4. CcO subunit I frameshift region. Alignment of 454 and Illumina reads with the frameshift region of cytochrome C oxidase subunit I. The combination of both technologies avoids the commom 454 homopolimer error, supporting the existence of the frameshift. First ten reads are from 454 sequencing. Last ten reads are from Illumina sequencing. S5. Gene clusters comprising B12 biosynthesis genes. The small cluster (red) harbors genes responsible for providing aminolaevulinic acid and converting it to uroporphyrinogen III, as well as the interconversion of uroporphyrinogen III into haem. The large cluster (green) harbors genes responsible for cobalt transport and adenosylcobalamin synthesis from uroporphyrinogen III. [file 1471-2164-13-562-S2.pdf]
