# Supplementary material for: The genome sequence of Propionibacterium acidipropionici provides insights into its biotechnological and industrial potential
Source: BMC Genomics. 2012 Oct 19;13:562. doi: 10.1186/1471-2164-13-562 (PMC3534718; doi:10.1186/1471-2164-13-562)
Supplement: Additional file 3 — Table S2. Transporter proteins comparison. Complete list of transporter families identified by annotation with TCDB classification. [file 1471-2164-13-562-S3.pdf]

TC system	*P. acidipropionici*	*P. acnes*	P. freudenreichii	*M. phosphovorus*
**1. Channels/Pores**	**11**	**15**	**9**	**22**
1.A.1.13	1	1	0	0
1.A.11.1	1	0	1	1
1.A.2.2	0	1	1	3
1.A.22.1	0	1	0	0
1.A.23.3	1	1	1	1
1.A.26.1	1	0	0	1
1.A.33.1	0	0	0	2
1.A.35.1	1	0	0	0
1.A.35.3	0	1	0	2
1.A.62.2	0	0	1	1
1.A.8.15	0	0	0	1
1.A.8.2	1	1	1	1
1.A.8.3	0	0	0	1
1.B.12.4	2	2	2	4
1.B.33.1	1	1	1	1
1.B.6.1	1	0	0	0
1.C.11.1	0	0	0	2
1.C.70.1	0	5	0	0
1.C.82.1	1	1	1	1
**2. Electrochemical Potential-driven Transporters**	**102**	**72**	**88**	**151**
2.A.1.1	8	5	5	8
2.A.1.12	0	2	0	0
2.A.1.14	0	1	2	1
2.A.1.15	0	1	0	2
2.A.1.17	0	0	0	1
2.A.1.18	1	0	1	0
2.A.1.2	2	0	2	8
2.A.1.20	0	0	0	1
2.A.1.21	2	0	2	3
2.A.1.3	10	3	12	21

2.A.1.30	2	1	2	2
2.A.1.36	1	0	1	0
2.A.1.4	0	0	2	0
2.A.1.40	1	1	1	1
2.A.1.46	1	1	1	1
2.A.1.52	0	1	1	0
2.A.1.53	0	1	0	0
2.A.1.59	0	0	0	1
2.A.1.6	7	2	4	10
2.A.1.60	1	0	0	4
2.A.1.67	0	0	0	1
2.A.1.7	0	1	0	1
2.A.1.8	2	1	0	2
2.A.101.1	0	0	0	1
2.A.102.3	1	1	0	1
2.A.102.6	1	0	0	1
2.A.11.1	0	0	0	1
2.A.13.1	0	2	2	0
2.A.14.1	1	1	1	1
2.A.15.1	0	0	0	1
2.A.16.2	1	1	0	0
2.A.17.1	0	1	0	1
2.A.19.1	0	0	1	0
2.A.2.2	1	0	0	0
2.A.2.3	2	1	1	0
2.A.2.5	1	0	0	0
2.A.2.6	0	0	0	1
2.A.20.1	1	1	0	2
2.A.20.2	0	0	1	1
2.A.21.3	1	1	1	0
2.A.21.9	5	3	3	8
2.A.22.4	0	1	0	0
2.A.23.1	1	0	0	1

2.A.25.1	0	1	0	0
2.A.26.1	0	1	0	0
2.A.28.2	1	0	0	0
2.A.3.1	9	11	7	2
2.A.3.14	1	1	1	1
2.A.3.2	0	1	0	0
2.A.3.3	2	0	0	2
2.A.3.4	1	0	0	0
2.A.3.5	0	0	0	1
2.A.3.6	0	0	2	1
2.A.3.7	0	0	1	0
2.A.33.1	3	1	1	2
2.A.36.3	1	0	1	1
2.A.36.6	1	2	1	1
2.A.37.1	0	0	0	1
2.A.37.4	0	0	0	1
2.A.39.1	0	0	1	1
2.A.39.2	0	1	0	0
2.A.39.3	1	0	0	1
2.A.4.1	1	1	1	4
2.A.4.6	1	1	1	2
2.A.4.7	1	0	1	0
2.A.40.1	1	1	1	0
2.A.40.3	0	0	1	1
2.A.42.2	0	1	0	0
2.A.45.2	1	0	0	1
2.A.47.3	0	0	2	0
2.A.47.4	0	0	0	1
2.A.52.1	0	0	0	1
2.A.53.4	0	0	0	4
2.A.53.5	0	0	0	1
2.A.53.9	0	0	0	1
2.A.55.2	1	0	1	0

2.A.55.3	0	1	0	0
2.A.59.1	0	0	0	2
2.A.59.2	0	0	1	0
2.A.6.2	0	1	0	1
2.A.6.5	2	1	2	1
2.A.63.1	0	0	0	3
2.A.64.1	1	0	0	0
2.A.64.3	0	1	1	1
2.A.66.1	2	1	0	0
2.A.66.2	2	0	0	0
2.A.66.4	1	0	1	2
2.A.67.4	1	1	1	0
2.A.7.1	1	0	1	1
2.A.7.17	1	0	0	0
2.A.7.26	0	0	0	1
2.A.7.3	1	1	1	4
2.A.7.3	0	0	0	1
2.A.7.7	0	0	0	1
2.A.72.1	1	1	1	0
2.A.75.1	1	0	1	0
2.A.76.1	0	0	0	1
2.A.78.1	1	0	0	1
2.A.79.1	0	1	0	1
2.A.8.1	1	1	2	1
2.A.80.1	0	0	0	2
2.A.81.1	1	1	1	0
2.A.86.1	1	1	2	3
2.A.88.1	0	0	0	1
2.A.89.3	1	1	1	2
2.A.9.2	0	0	1	0
2.A.9.3	1	1	0	1
2.A.93.1	1	1	1	0
2.A.98.1	1	0	1	1

3. Primary active transporters	281	193	158	285
3.A.1.1	57	29	7	40
3.A.1.10	0	0	0	4
3.A.1.102	2	1	0	1
3.A.1.103	0	0	2	2
3.A.1.104	0	0	0	1
3.A.1.105	4	1	4	6
3.A.1.106	3	2	3	10
3.A.1.11	1	1	0	1
3.A.1.111	0	0	0	3
3.A.1.119	0	2	1	8
3.A.1.12	13	6	7	6
3.A.1.120	4	2	3	4
3.A.1.121	1	1	1	1
3.A.1.122	3	1	1	10
3.A.1.124	1	0	0	1
3.A.1.125	7	7	4	3
3.A.1.126	3	0	1	1
3.A.1.128	2	1	2	1
3.A.1.129	2	3	1	1
3.A.1.131	1	1	1	2
3.A.1.132	4	5	2	1
3.A.1.134	3	1	0	2
3.A.1.135	2	1	1	2
3.A.1.136	0	1	0	0
3.A.1.139	0	0	0	1
3.A.1.14	6	17	9	18
3.A.1.141	1	0	0	3
3.A.1.15	4	6	3	6
3.A.1.16	3	0	0	0
3.A.1.17	2	0	2	4
3.A.1.18	1	2	2	0
3.A.1.19	1	0	0	0

3.A.1.2	17	7	4	9
3.A.1.20	0	0	0	4
3.A.1.201	0	0	1	0
3.A.1.204	1	0	0	0
3.A.1.205	1	0	0	1
3.A.1.21	2	2	1	2
3.A.1.210	2	0	0	1
3.A.1.23	1	1	0	0
3.A.1.24	3	3	3	0
3.A.1.25	1	2	1	1
3.A.1.27	0	1	0	0
3.A.1.3	19	5	8	11
3.A.1.30	0	2	2	0
3.A.1.31	0	1	0	3
3.A.1.32	3	3	3	0
3.A.1.34	0	0	3	0
3.A.1.4	5	0	5	5
3.A.1.5	28	14	9	29
3.A.1.6	0	0	4	4
3.A.1.7	4	4	4	4
3.A.1.8	2	1	0	2
3.A.10.1	0	1	0	0
3.A.10.2	1	0	0	1
3.A.11.1	0	2	2	2
3.A.12.1	1	1	1	1
3.A.16.1	1	1	1	2
3.A.17.1	0	0	1	0
3.A.18.1	2	2	2	3
3.A.2.1	3	3	4	3
3.A.20.1	0	0	1	1
3.A.3.1	2	2	2	2
3.A.3.2	1	0	0	0
3.A.3.23	0	0	0	1

3.A.3.5	3	2	3	3
3.A.3.6	2	0	1	3
3.A.3.7	0	3	0	3
3.A.4.1	1	0	1	0
3.A.5.2	5	5	6	5
3.A.6.1	1	1	1	1
3.A.7.11	1	1	0	2
3.A.7.13	0	0	0	1
3.A.7.15	2	2	2	2
3.A.7.7	1	1	1	0
3.A.9.1	1	2	2	2
3.B.1.1	7	3	4	4
3.D.1.1	2	1	0	2
3.D.1.2	2	4	4	2
3.D.1.3	5	4	4	4
3.D.1.5	2	3	2	2
3.D.1.6	2	2	2	2
3.D.1.7	0	0	0	1
3.D.2.1	2	2	2	1
3.D.2.2	0	2	1	2
3.D.2.3	1	0	0	1
3.D.4.10	2	1	1	1
3.D.4.3	1	0	1	1
3.D.4.4	2	3	0	3
3.D.4.5	1	1	0	1
3.D.5.1	1	0	0	0
3.D.6.1	1	0	0	0
3.D.9.1	1	0	1	0
3.E.2.2	1	1	0	1
**4. Group translocators**	**20**	**28**	**6**	**9**
4.A.1.1	0	6	0	0
4.A.1.2	1	2	1	0
4.A.2.1	2	4	0	0

4.A.3.2	0	3	0	0
4.A.4.1	5	4	0	0
4.A.5.1	4	2	0	0
4.A.6.1	0	0	1	0
4.A.7.1	0	2	0	0
4.B.1.1	1	1	0	0
4.C.1.1	7	4	4	9
**5. Transmembrane electron carriers**	**8**	**11**	**7**	**10**
5.A.1.2	0	1	0	1
5.A.3.1	3	3	0	3
5.A.3.2	0	0	0	3
5.A.3.3	0	3	2	0
5.A.3.4	0	0	0	1
5.A.4.1	5	4	5	2
**8. Accessory Factors involved in Transport**	**13**	**9**	**10**	**25**
8.A.1.1	0	0	0	1
8.A.21.2	2	2	1	2
8.A.28.1	0	0	0	1
8.A.3.2	1	0	0	0
8.A.5.1	4	3	5	13
8.A.7.1	2	2	2	2
8.A.9.1	4	2	2	6
**9. Incompletely characterized**	**34**	**16**	**23**	**30**
9.A.10.1	1	0	1	2
9.A.10.2	4	0	1	3
9.A.19.1	1	0	1	1
9.A.25.1	1	0	0	2
9.A.30.1	2	1	2	1
9.A.34.2	2	2	3	2
9.A.40.1	1	1	1	1
9.A.40.2	3	1	1	1

9.A.41.1	1	0	0	0
9.A.44.1	0	0	0	1
9.A.49.1	0	0	2	0
9.A.8.1	1	1	1	0
9.B.10.1	1	0	0	1
9.B.102.4	0	0	1	1
9.B.20.1	2	0	0	0
9.B.27.2	3	1	1	1
9.B.3.1	2	2	2	2
9.B.30.1	1	0	1	1
9.B.32.1	1	0	0	2
9.B.35.2	0	0	0	1
9.B.42.1	0	0	0	1
9.B.45.1	2	2	2	3
9.B.50.1	0	1	0	0
9.B.59.1	1	1	1	0
9.B.74.1	2	2	1	2
9.B.74.2	1	0	0	0
9.B.76.1	1	1	1	1
