# Supplementary material for: The genome sequence of Propionibacterium acidipropionici provides insights into its biotechnological and industrial potential
Source: BMC Genomics. 2012 Oct 19;13:562. doi: 10.1186/1471-2164-13-562 (PMC3534718; doi:10.1186/1471-2164-13-562)
Supplement: Additional file 4 — Table S3. PTS protein-coding genes. List of annotated genes related to phosphotransferase system in P.acidipropionici genome. [file 1471-2164-13-562-S4.pdf]

Additional file 4. PTS protein-coding genes: list of annotated genes related to phosphotransferase system in *P.acidipropionici* genome.

**Locus tag**	**PTS related enzymes**
PACID_08180	Glucitol/sorbitol-specific enzyme IIC
PACID_08190	Glucitol/sorbitol-specific enzyme IIB
PACID_08200	Glucitol/sorbitol-specific enzyme IIA
PACID_21740	PRD domain protein
PACID_21750	Sorbitol operon activator
PACID_21760	Glucitol/sorbitol-specific enzyme IIC
PACID_21770	Glucitol/sorbitol-specific enzyme IIB
PACID_21780	Glucitol/sorbitol-specific enzyme IIA
PACID_19180	Glucose-specific enzyme IIABC
PACID_14130	Manitol-specific enzyme IIABC
PACID_04770	Fructose-specific enzyme IIABC
PACID_26550	Dihydroxyacetone K subunit
PACID_26560	Dihydroxyacetone L subunit
PACID_26570	Dihydroxyacetone phosphotransferase subunit
PACID_00890	Sugar-specific enzyme IIA
PACID_00920	Galactitol-specific enzyme IIC
PACID_00910	Lactose/celobiose-specific enzyme IIB
PACID_08140	Lactose/celobiose-specific enzyme IIB
PACID_08130	Galactitol-specific enzyme IIC
PACID_00930	Galactitol-specific enzyme IIA
PACID_04760	Hpr enzyme
PACID_08030	Hpr enzyme
PACID_21790	Hpr enzyme
